# Phytochemical Constituents of Selected *Garcinia* Fruits from Tropical Australia

**DOI:** 10.3390/foods15203720

**Published:** 2026-10-19

**Authors:** Oliver D. John, Sunil K. Panchal, Lindsay Brown, Peter Mouatt

**Affiliations:** 1 Functional Foods Research Group, University of Southern Queensland, Toowoomba, QLD 4350, Australia; odjohn@ums.edu.my (O.D.J.); s.panchal@westernsydney.edu.au (S.K.P.); lindsay.brown@griffith.edu.au (L.B.); 2 Analytical Research Laboratory, Southern Cross Plant Science, Southern Cross University, East Lismore, NSW 2480, Australia

**Keywords:** *Garcinia humilis*, *Garcinia mangostana*, *Garcinia dulcis*, *Garcinia gummi-gutta*, organic acid, flavonoids, anthocyanins, procyanidin, xanthone, benzophenone, morelloflavone

## Abstract

*Garcinia* species grow well in tropical regions and have been widely consumed, especially among local communities. *Garcinia* fruits and their byproducts contain a range of bioactive compounds that have potential to work as nutraceuticals or functional foods. We have determined the phytochemical constituents of rind, pulp and seeds from *G. humilis*, *G. mangostana*, *G. dulcis* and *G. gummi-gutta* fruits grown in tropical Queensland, Australia. We used Liquid Chromatography–Mass Spectrometry to define the variations between species in the content of citric acid, hydroxycitric acid, benzophenones, garcinols, flavonoids, biflavonones, tocotrienols and other compounds. *G. humilis* rind has mainly citric acid (12.14% *w*/*w*) and procyanidin B_2_ (6% *w*/*w*), with the seeds containing guttiferone derivatives (3.56% *w*/*w*) and garcinol (2.69% *w*/*w*). *G. mangostana* rind contained xanthones (6.49% *w*/*w*; α–mangostin 5.42% *w*/*w*), procyanidin B_2_ (12.6% *w*/*w*), cyanidin glycoside (0.137% *w*/*w*) and hydroxycitric acid (0.42% *w*/*w*). The rind of *G. dulcis* contained benzophenones (7.86% *w*/*w*; garcinol: 6.35% *w*/*w*), citric acid (24% *w*/*w*) and morelloflavone (0.55% *w*/*w*); the seeds contained morelloflavone (2.9% *w*/*w*) and xanthone derivatives. *G. gummi-gutta* rind contained hydroxycitric acid (28.8% *w*/*w*) and citric acid (3.76% *w*/*w*/), whereas pulp contained citric acid (5.74% *w*/*w*) and seeds contained morelloflavone. *G. humilis*, *G. dulcis* and *G. mangostana* have more complex phytochemical constituents compared to *G. gummi-gutta*. This study showed that fruits from *Garcinia* species from tropical Australia are rich sources of phytochemicals that can be processed for the nutraceutical and food industries.

## 1. Introduction

The genus *Garcinia* belongs to the Clusiaceae or Guttiferae family and contains more than 400 species worldwide [1,2,3]. The trees are evergreen and grow in lowland tropical forests [3]. The juicy pulp of the fruit ranges from white to pinkish to orange in colour, and mostly the fruits have a tangy, sour to sweet flavour [4,5]. Due to their flavours, *Garcinia* fruits have been used for culinary purposes in Asian countries, with the fruit endocarp described as a subtle delicacy [6,7]. *G. mangostana*, or mangosteen, is widely grown commercially in Southeast Asia, with Thailand producing around 545,000 metric tonnes/year [8]. In Australia, mangosteen is grown in north Queensland for a small niche market with annual production around 200 tonnes and an estimated industry value of AU$1–3 million [9]. *G. humilis*, also known as achacha, achachairú, or Bolivian mangosteen, is now grown commercially in Australia [10]. It is gaining popularity as a dessert fruit [11,12]. *Garcinia* fruits have been used in traditional medicine as an expectorant to clear sputum, alleviate cough, treat scurvy, and as a mild laxative [13,14,15].

*Garcinia* plants contain many phytochemicals including polyphenols such as oxygenated and prenylated xanthones, prenylated benzophenones such as guttiferones, as well as flavonoids, anthocyanins and procyanidins [1,16,17,18]. Benzophenones are non-polar phenolic molecules, having prenyl functional groups that are the main intermediates in xanthone biosynthetic pathways [19]. *G. gummi-gutta*, previously *G. cambogia*, fruit contains phytochemicals including hydroxycitric acid, xanthones and benzophenones [13,16,20]. The major xanthones in *G. mangostana* pericarp are α-mangostin and γ-mangostin [15,21]. Phenolic compounds including dulcisxanthones A-B, dulcinoside, dulcisisoflavone and dulcisflavan were isolated from the green fruit of *G. dulcis* [22]. The phytochemicals in the fruit and rind of *G. dulcis* have been discussed [23], but information about *G. humilis* is still restricted to the rind and pulp. Further, extension of the preliminary studies on the phytochemicals of these *Garcinia* fruits grown in Australia [12,24,25] may allow analysis of possible changes in phytochemical constituents of a fruit due to geographical distribution [26].

Overall, previous phytochemical characterisation of *Garcinia* species focused on specific fruit tissues and selected phytochemical classes. A comparative analysis of the phytochemical composition of pulp, rind and seed across multiple *Garcinia* species will help define the phytochemical profile of these fruits, especially the seed and rind as byproducts. Thus, this study characterised the phytochemicals from the fruit pulp, rind and seed of *G. mangostana*, *G. gummi-gutta*, *G. dulcis* and *G. humilis* sourced from tropical Queensland. This information may then allow wider research on these components of these tropical fruits for their possible therapeutic benefits in chronic diseases. As rind and seeds are usually discarded, these studies may also allow growers to expand their business options to develop functional foods from food byproducts.

## 2. Materials and Methods

### 2.1. Plant Material

**Figure 1:**
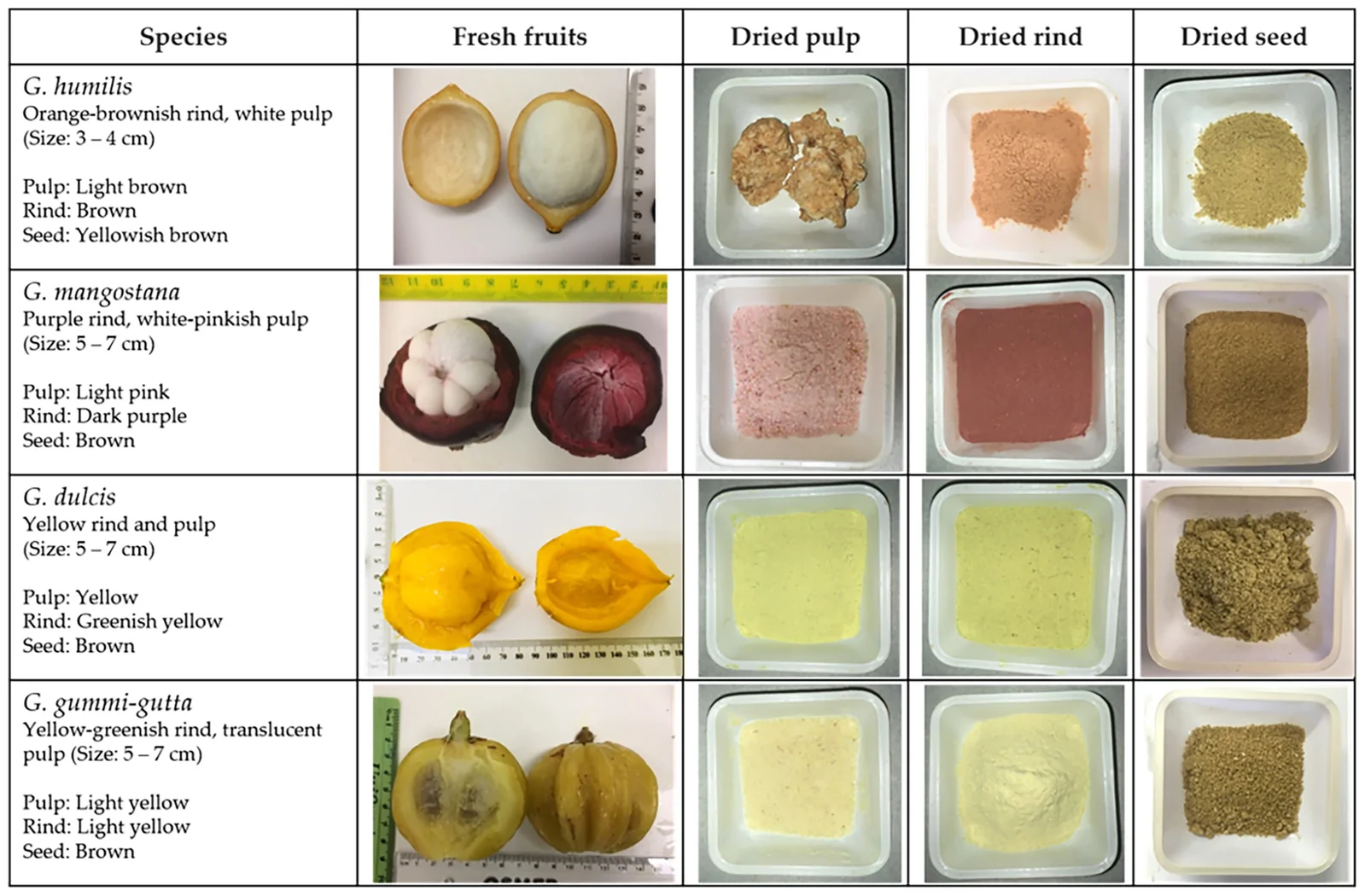
Physical characteristics of fresh fruit and freeze-dried products of *G. humilis*, *G. mangostana*, *G. dulcis* and *G. gummi-gutta*.

*G. humilis* fruit was supplied by Achacha Fruit Group, Giru, QLD, Australia. *G. mangostana* was grown in Tully, QLD, Australia. *G. dulcis* was kindly donated by Mr. Neil Wiltshire, horticulturist from the Department of Agriculture, South Johnstone, QLD, Australia. *G. gummi-gutta* was kindly donated by Mr. David Warmington, curator at the Cairns Botanical Garden, Cairns, QLD, Australia. The ripe fruits were separated into rind, pulp and seed. The rind, seed and pulp were stored at –20 °C, freeze-dried, and ground into powder form (Figure 1). The freeze-dried powder was later used for chemical analysis.

### 2.2. Chemicals and Reagents

The chemicals and reagents used in this study comprised the following: Ethanol (Scharlau (Barcelona, Spain), Product Code E100154000), acetonitrile (Scharlau, HPLC grade, Product Code AC03314000), trifluoroacetic acid (Sigma-Aldrich (Bayswater, VIC, Australia), Cat No T6508), sodium acetate (Sigma-Aldrich), sodium carbonate (Sigma-Aldrich), potassium dihydrogen phosphate (BDH (Poole, England), Product Code 10203.4B 27594), phosphoric acid (Sigma-Aldrich, Product Code 04107), formic acid (Scharlau, Cat No AC10760050), hydrochloric acid (Sigma-Aldrich, Product Code 102210458), methanol (LabSolv (Bangkok, Thailand), Product Code 03S0014104), Folin–Ciocalteu reagent (Merck (Bayswater, VIC, Australia), Product Code 1.09001), gallic acid (Sigma-Aldrich, Cat No G7384), catechin (Sigma-Aldrich, Cat No C1251), phloroglucinol (Sigma Aldrich, Cat No P3502), procyanidin B_2_ (Phytolab (Vestenbergsgreuth, Germany), Cat No 89552 6783), hydroxycitric acid calcium salt (Chromadex (Irvine, CA, USA), Cat No. 00008386), citric acid (Sigma-Aldrich, Cat No. 240621), malic acid (Sigma-Aldrich, Cat No. 240176), cyanidin 3-glucoside chloride (Phytolab, Cat No 80022 5368), rutin (Sigma-Aldrich, Cat No R5143), quercetin (Sigma-Aldrich, Cat No Q0125), α-mangostin (Indofine Chemical Company Inc. Hillsborough, NJ, USA, Cas No. 6147-11-1, Cat No. M002), garcinol (Sigma-Aldrich, Cas No. 78824-30-3, Cat No. G5173) and naringin (Sigma-Aldrich, Cat No. N5893).

### 2.3. Total Phenolic Content

The total phenolic content was determined using the method of Singleton and Rossi [27] with modifications. For phenolic content extraction, 0.5 g of freeze-dried rind, pulp and seed samples from each fruit were separately extracted with 10 mL of 50:50 ethanol/water solution in a horizontal shaker (200 rpm) for 2 h at room temperature. The mixture was separated by centrifugation at 4000 rpm for 30 min. The supernatant was further used for phenolic acid analysis. Total phenolic content was analysed by reacting 100 µL of collected supernatant with 2.5 mL of 10% Folin–Ciocalteu reagent. After 4 min, 2 mL of 7.5% *w*/*v* sodium carbonate solution was added. The mixture was incubated at room temperature for 60 min in the dark and absorbance was measured at 760 nm using only solvent as the blank. Gallic acid was used as the standard for the calibration curve, and the results were expressed as mg of gallic acid equivalents (mg GAE)/g of dry weight (dw). Two-way ANOVA was used to assess the differences in total phenolic content values between different fruit parts and between different fruit species. Significant main effects were further evaluated using Tukey’s HSD post hoc test. A *p*-value of less than 0.05 was considered statistically significant.

### 2.4. LC-MS Analysis

The freeze-dried extracts were analysed by Liquid Chromatography–Mass Spectrometry (LC-MS) using an Agilent 1200 series High-Performance Liquid Chromatography (HPLC) system with diode array detection (DAD) from 190 to 800 nm coupled with an Agilent single quad mass spectrometer (Agilent Technologies Australia, Mulgrave, VIC, Australia). The mass spectral analysis was performed with atmospheric pressure chemical ionisation operated in positive mode with scanning from 100 to 1500 atomic mass units. The chromatography was performed on a Phenomenex Luna C18 HPLC column (100 × 4.6 mm, Phenomenex Australia Pty Ltd., Lane Cove, NSW, Australia) (unless stated differently) using Milli-Q water and acetonitrile with 0.005% trifluoroacetic acid. The method involved a gradient of 10% acetonitrile, 90% water to 95% acetonitrile, 5% water over 18 min, at a flow rate of 0.75 mL/min.

Peaks were tentatively identified based on UV–visible and mass spectra and comparison with literature reports and previous phytochemical analysis, where possible, by comparison with reference standards such as catechin. When standards were unavailable, compounds were identified using UV–visible and/or absorbance maxima by comparison with the published Natural Products database [28]. When good MS ions were not available, broad identification of some peaks was based on common and characteristic UV–visible spectra.

### 2.5. Procyanidin HPLC Analysis

The quantification of procyanidins in the sample was performed by HPLC using a method developed for analytical purposes based on previously published methods using hydrolysis and derivatisation of the flavanol units to quantify the varying polymeric forms of procyanidins [29]. Briefly, hydrolysis of the B-type procyanidins, with single bonds between each flavanol unit, in the presence of the nucleophile phloroglucinol, forms reaction products with each flavanol unit except the terminal unit of the procyanidin. The range of polymeric procyanidins was converted into a series of common products, which were quantified as dimeric procyanidin B_2_.

The hydrolysis reagent solution consisted of 0.2 M HCl containing 10 mg/mL phloroglucinol and 10 mg/mL ascorbic acid. A 160 mM solution of sodium acetate was prepared for quenching hydrolysis. Briefly, freeze-dried samples were extracted into a 50:50 ethanol/water mixture with sonication for 15 min. One aliquot (0.5 mL) was taken and diluted to 7.5 mL with ethanol/water to determine free flavanol content. Another 0.5 mL aliquot was placed with 2 mL of phloroglucinol reagent in a reaction tube and heated in a water bath at 78 °C for 60 min. After cooling, 5 mL of the sodium acetate solution was added to quench the hydrolysis reaction, with a final volume of 7.5 mL. Aliquots of both the diluted and hydrolysed samples were placed in a mini vial for HPLC analysis.

For quantification, a catechin reference standard was prepared as a serial dilution from 1 to 0.01 mg/mL in 50:50 ethanol/water (Table 1). The dimeric procyanidin B_2_ was also prepared as a serial dilution from 5 to 0.05 mg/mL. Then 0.5 mL aliquots were taken and diluted or hydrolysed with the phloroglucinol reagent as per the test sample. A calibration curve of the hydrolysis products of procyanidin B_2_ was used to calculate the procyanidin content of the test sample. Free flavanols were calculated using catechin for the unhydrolysed sample and total procyanidins were calculated from the hydrolysed sample.

### 2.6. Organic Acid Analysis

Analysis of organic acids was based on the 2016 USP *Garcinia* hydroxycitric acid method [30]. All reagents and solvents used were HPLC grade. The mobile phase was 0.136% *w*/*v* potassium dihydrogen phosphate in 3% phosphoric acid adjusted to pH 2.5.

Briefly, about 250 mg of the extracts were weighed and extracted in 5 mL of 3% phosphoric acid with sonication for 15 min. After centrifuging for 5 min, an aliquot of the supernatant was taken up in an HPLC vial and run against reference standards of hydroxycitric acid calcium salt, citric acid and malic acid.

The analysis was performed on an Agilent 1200 series HPLC, using a Phenomenex 250 mm C18 column with 1 mL/min of isocratic mobile phase over 25 min. Reference standards were injected and prepared as a calibration curve for calculation of the hydroxycitric acid, malic acid, quinic acid and citric acid concentrations. For the hydroxycitric acid profile, the rind of *G. quaesita* was held as a reference sample at the Analytical Research Laboratory, Southern Cross University, for herbal authentication.

The freeze-dried powders were analysed on an Agilent 1200 series HPLC system consisting of a diode array detector (G4212B), binary pump (G4220A), an autosampler (G4226A), a vacuum degasser and a column oven. All solvents and reagents used were HPLC- or analytical-grade.

### 2.7. Anthocyanin Analysis

The chromatography was performed with a Phenomenex Luna C18 HPLC column (250 × 4.6 mm) using a gradient method as described in the BP2016 for analysis of anthocyanin content in bilberry extracts monograph [31]. The mobile phases were solvent A (8.5% formic acid, Milli-Q water) and solvent B (8.5% formic acid, 22.5% acetonitrile, 22.5% methanol). The gradient started at 7% solvent B, increased to 25% over 35 min, then to 65% solvent B over 10 min, at a flow rate of 1 mL/min and an injection volume of 10 µL. Specific detection and calibration for each compound were performed at 535 nm.

Solvents for reference and sample preparation were 2% hydrochloric acid in methanol and dilute (0.1%) phosphoric acid. Calibration standards of cyanidin 3-glucoside chloride (10 mg) and a standardised bilberry extract (3.34% cyanidin 3-glucoside) (125 mg) were prepared in acidified methanol (25 mL), then diluted in dilute phosphoric acid, 2 mL into 100 mL and 5 mL into 20 mL, respectively. Sample extracts were weighed and extracted in acidic methanol (25 mL), sonicated for 15 min, then centrifuged. An aliquot (2.5 mL) of the supernatant was then diluted with dilute phosphoric acid up to 10 mL, equilibrated for 15 min, and then an aliquot was taken into an HPLC vial for analysis. Quantification was performed as described in the BP2016 based on reference standards, peak area at 535 nm, and sample dilution. Total anthocyanins were calculated as cyanidin 3-glucoside and expressed as mg/100 g dw.

### 2.8. Flavonoid Analysis

The chromatography was performed with a Phenomenex Luna C18 HPLC column (250 × 4.6 mm) using a gradient method as described in the USP2016 *Ginkgo biloba* extract monograph for analysis [32] and limits of rutin and quercetin content. The mobile phases were solvent A (0.1% formic acid, Milli-Q water) and solvent B acetonitrile over 45 min. The gradient started at 10% solvent B, which was increased to 36% solvent B over 40 min, then to 100% solvent B over 5 min, followed by a 5 min washout and return to initial conditions over 10 min, with a flow rate of 1 mL/min. Specific detection and calibration for each compound were performed at 254 nm.

Reference standards of rutin and quercetin were prepared in methanol at 0.59 mg/mL and 0.142 mg/mL, respectively, then diluted for a five-point calibration curve. Sample extracts were weighed out and extracted in methanol (25 mL), sonicated for 15 min, then centrifuged. An aliquot (2.5 mL) of the supernatant was then taken into an HPLC vial for analysis. Quantification was performed based on a calibration curve of reference standards, peak area at 254 nm, and sample dilution. Total flavonoid glycosides were calculated as rutin, quercetin was calculated as quercetin, and results were expressed as mg/100 g dw.

### 2.9. Xanthone (α-Mangostin) Analysis

The chromatography was performed with a Phenomenex Kinetex C18 HPLC column (100 × 4.6 mm) using a gradient method based on a previously published method [33]. Freeze-dried ground fruit part was quantitatively extracted by mixing 0.2 to 0.25 g with 25 mL of acetonitrile in a volumetric flask and sonicated for 30 min. An aliquot of the solvent sample was centrifuged and 200 µL of the supernatant was taken into an HPLC vial for analysis. The mobile phases were solvent A (0.1% formic acid, Milli-Q water) and solvent B (acetonitrile with 0.1% formic acid); optimal separations were obtained using a gradient of 60–95% B over 0–28 min, with a flow rate of 0.75 mL/min. Specific detection and calibration for each xanthone compound were performed at 240 nm.

A reference standard of α-mangostin was prepared in acetonitrile at 0.4 mg/mL, then diluted to produce a five-point calibration curve. Quantification was performed based on the calibration curve of reference standards, peak area at 240 nm, and sample dilution. Total xanthones were calculated as α-mangostin and results were expressed as % *w*/*w* dw.

### 2.10. Benzophenone (Garcinol) Analysis

The method for benzophenone compound analysis was identical to the xanthone method described in Section 2.8, but using garcinol as standard. The reference standard of garcinol was prepared in acetonitrile at 0.4 mg/mL, then diluted to produce a five-point calibration curve. Quantification was performed based on the calibration curve of reference standards, peak area at 240 nm, and sample dilution. Total benzophenones were calculated as garcinol and results were expressed as % *w*/*w* dw.

### 2.11. Morelloflavone Analysis

The chromatography was performed on a Phenomenex luna C18 HPLC column (100 × 4.6 mm) using a gradient method of water and acetonitrile with 0.005% trifluoroacetic acid over 28 min. The solvent gradient for separation of target constituents commenced at 10% acetonitrile and increased as a gradient to 95% acetonitrile over 18 min, at a flow rate of 0.75 mL/min and an injection volume of 5 μL. Morelloflavones are biflavones of naringin (flavanone) and luteolin (flavone) units; hence, naringin was used as the reference standard for calculating the quantity of morelloflavones. Calibration standards of naringin were prepared in water, at concentrations from 0.01 to 0.4 mg/L. Specific detection and calibration curves for each compound were performed at 280 nm. Quantification was performed using the ChemStation software Revision C.01.10 (Agilent Technologies Australia) based on reference standards, peak area and sample dilution at 280 nm for each peak identified as a biflavonone or glycoside. Total morelloflavone was calculated as naringin and results were expressed as % *w*/*w* of dw.

### 2.12. Chlorogenic Acid Analysis

For analysis, extracts were prepared at 200 mg freeze-dried powder per 10 mL water, and an aliquot of the supernatant was taken for HPLC analysis using an Agilent 1200 series with a diode array detector, on a Phenomenex luna C18 HPLC column (100 × 4.6 mm) using a gradient method of water and acetonitrile with 0.005% trifluoroacetic acid over 28 min. The optimal solvent gradient for separation of target constituents started at 10% acetonitrile, which was increased as a gradient to 30% acetonitrile over 10 min, then to 95% acetonitrile over 8 min, at a flow rate of 0.75 mL/min and an injection volume of 5 μL. Calibration standards of chlorogenic acid were prepared in water, at concentrations from 0.004 to 0.4 mg/mL. Specific detection and calibration curve were performed at 330 nm.

### 2.13. Chemical Structures

Chemical structures were drawn using ACD/ChemSketch Freeware, version 2023.1.2 (Advanced Chemistry Development Inc., Toronto, ON, Canada).

## 3. Results

### 3.1. Physical Appearance

The physical appearance of the fruits is summarised in Figure 1. The freeze-dried products of different fruit parts of *G. humilis*, *G. mangostana*, *G. dulcis* and *G. gummi-gutta* showed different colours and characteristics according to the species (Figure 1).

### 3.2. Total Phenolic Content

The total phenolic contents of the samples from *G. humilis*, *G. mangostana*. *G. dulcis* and *G. gummi-gutta* are summarised in Table 2. Total phenolic content was the highest in *G. mangostana* rind, followed by *G. gummi-gutta* seed and *G. humilis* rind, whereas the lowest total phenolic content was found in *G. gummi-gutta* rind.

### 3.3. LC-MS Analysis

Detailed profiles of the phytochemical constituents are given in Table 3, Table 4 and Table 5 and the Supplementary Materials (Supplementary Figures S1–S24) with structures in Figure 2.

#### 3.3.1. *G. mangostana*

**Figure 3:**
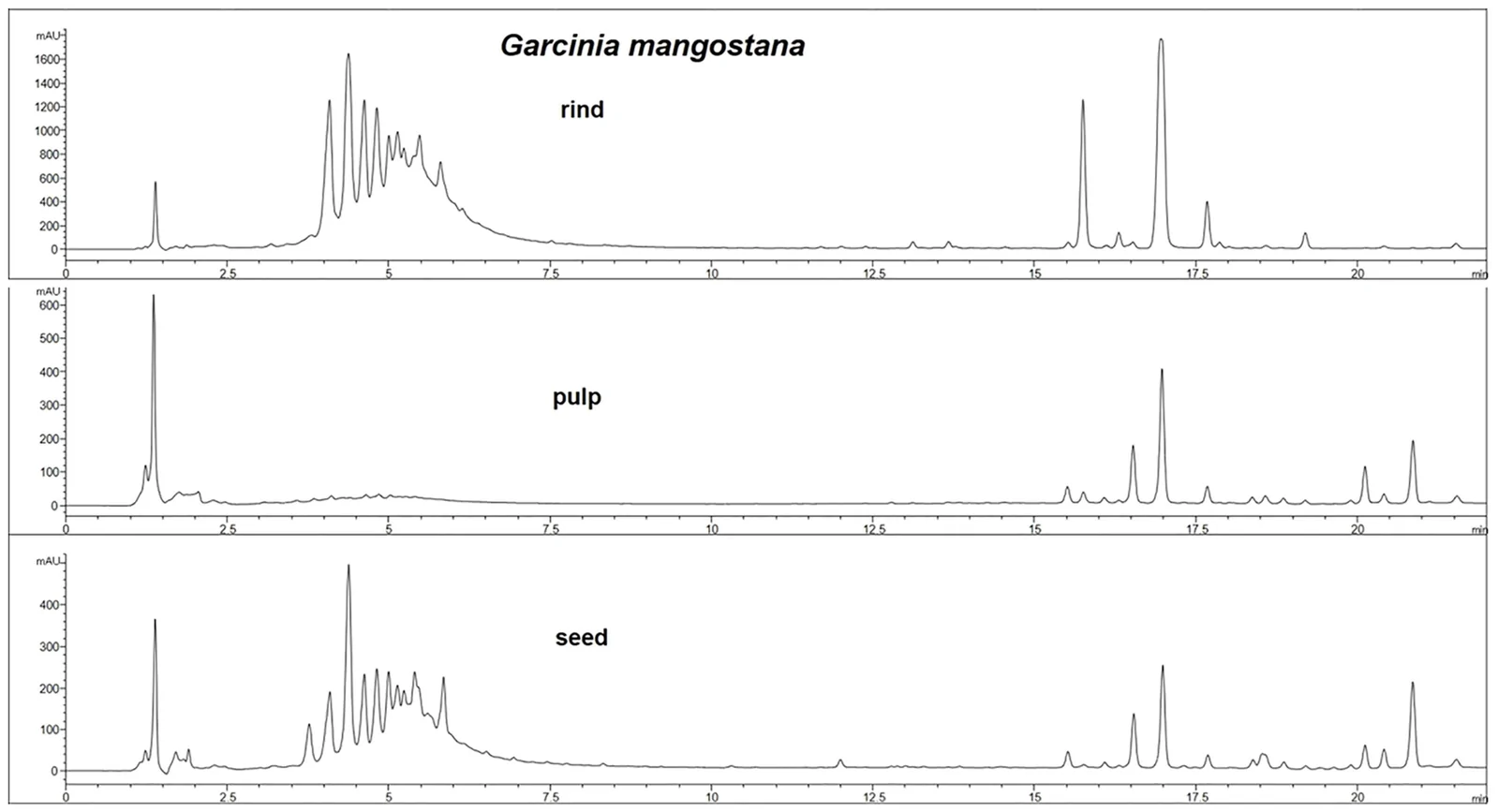
Liquid Chromatography–Mass Spectrometry chromatograms for *G. mangostana* rind, pulp and seed.

The rind of *G. mangostana* primarily contains anthocyanins, flavanols, procyanidins, xanthone derivatives and garcinone E. Of the xanthones, α-mangostin was the major compound (5.42% *w*/*w* dw, Table 3). Similarly, *G. mangostana* pulp contains xanthone derivatives and garcinone E but in lower abundance (Table 4). The α-mangostin pulp concentration was 0.087% *w*/*w*. In addition, the pulp also contains parvifoliol E and tocotrienic acid aldehyde (Table 4). The seed is more complex, with the presence of chlorogenic acid, procyanidins, phenolic compounds, xanthone derivatives, parvifoliol E and F, tocotrienic acid aldehyde and garcinone E (Table 5). LC-MS chromatograms for *G. mangostana* rind, pulp and seed are presented in Figure 3. Further details of the LC-MS chromatogram and UV–visible spectra can be found in Supplementary Figures S1–S3.

#### 3.3.2. *G. humilis*

**Figure 4:**
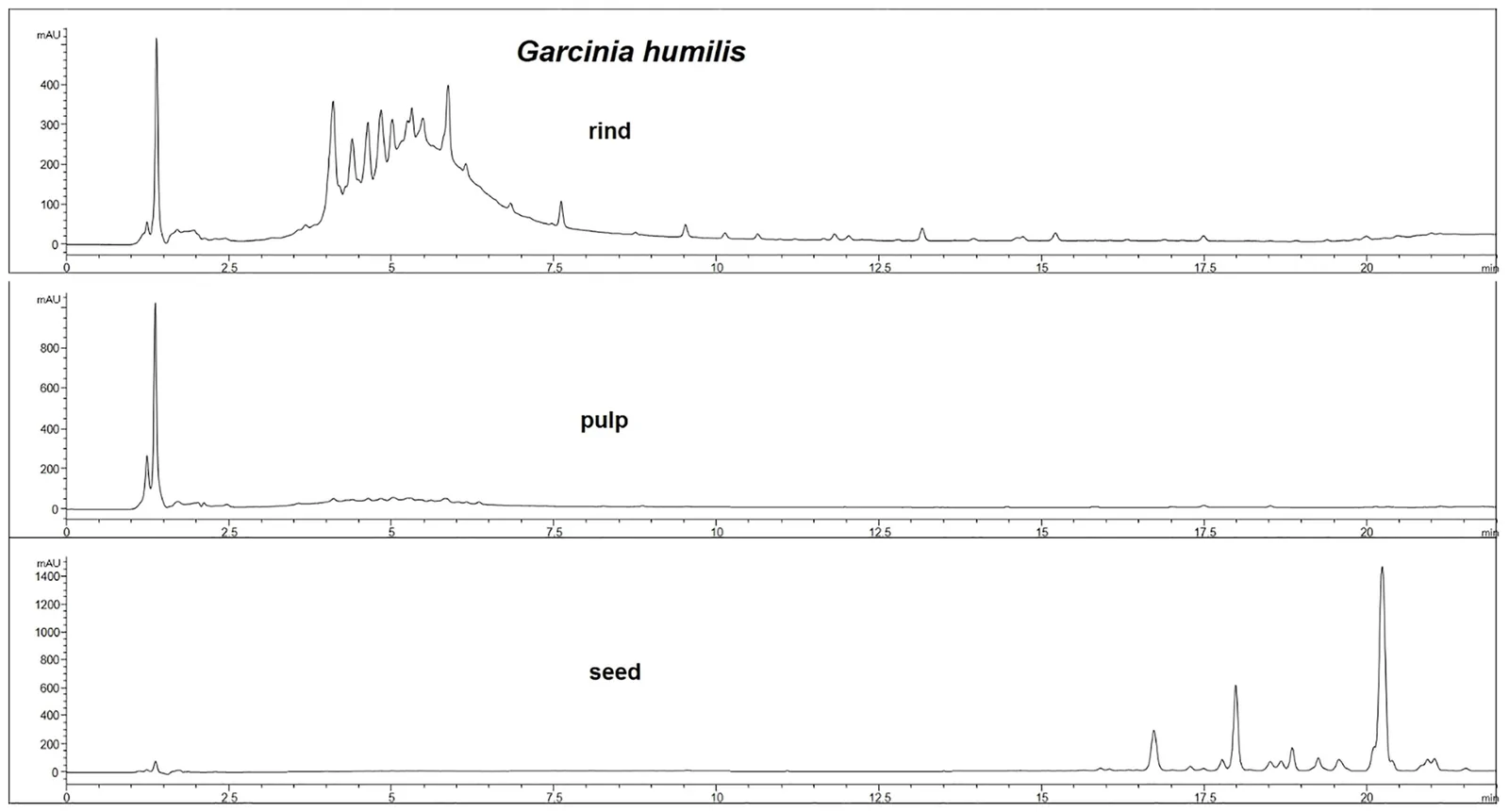
Liquid Chromatography–Mass Spectrometry chromatograms for *G. humilis* rind, pulp and seed.

The compounds identified in the rind of *G. humilis* included citric acid (12.14% *w*/*w* dw), hydroxycitric acid, flavan-3-ols, catechin and epicatechin, procyanidins A_2_, B_1_ and B_2_, flavone C-glycosides, flavanone and bi-flavanone glycosides, xanthone derivatives and depsidone (Table 3) [12]. The yield of procyanidins was about 6% *w*/*w* of freeze-dried rind powder but about 0.32% *w*/*w* of freeze-dried pulp powder. Similarly, the *G. humilis* freeze-dried pulp contains citric acid (1.44% *w*/*w* dw), hydroxycitric acid, flavan-3-ol, procyanidins, flavone C-glycosides, flavone derivatives and xanthone derivatives (Table 4). The seed has fewer compounds, mainly guttiferone derivatives, isogarcinol, and garcinol or garcinol-like compounds (Table 5). LC-MS chromatograms for *G. humilis* rind, pulp and seed are presented in Figure 4. Further details of the LC-MS chromatogram and UV–visible spectra can be found in Supplementary Figures S4–S6.

#### 3.3.3. *G. dulcis*

**Figure 5:**
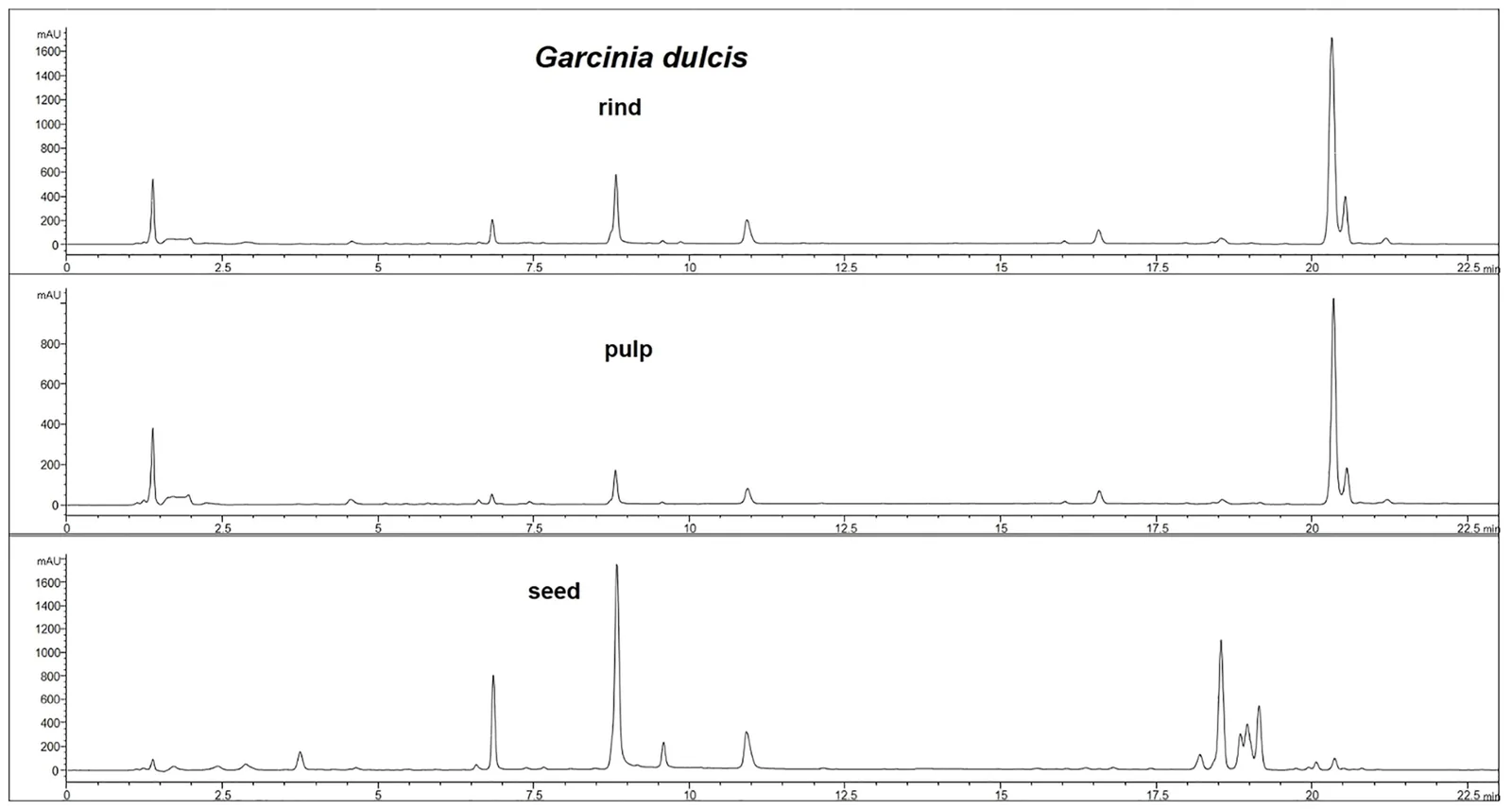
Liquid Chromatography–Mass Spectrometry chromatograms for *G. dulcis* rind, pulp and seed.

Garcinol constitutes about 80% of the benzophenone content in both the rind and pulp. The garcinol content was 6.35% *w*/*w* in the *G. dulcis* rind powder and 2.87% *w*/*w* in the *G. dulcis* pulp powder (Table 3 and Table 4). The contents of citric acid in the freeze-dried rind and pulp were 24% *w*/*w* and 19% *w*/*w*, respectively; however, no hydroxycitric acid was detected (Table 3 and Table 4). The rind, pulp and seeds of *G. dulcis* contained coumaric acid and the biflavone, morelloflavone (Table 3, Table 4 and Table 5). The *G. dulcis* seed was similar to *G. gummi-gutta* but different from other *Garcinia* in that it contained benzylamine derivatives, tocotrienic acid aldehyde, and xanthone derivatives (Table 5). The freeze-dried seeds have morelloflavone (2.9% *w*/*w*) and diprenyl-xanthone derivatives, but citric acid and benzophenones were not detected. LC-MS chromatograms for *G. dulcis* rind, pulp and seed are presented in Figure 5. Further details of the LC-MS chromatogram and UV–visible spectra can be found in Supplementary Figures S7–S9.

#### 3.3.4. *G. gummi-gutta*

**Figure 6:**
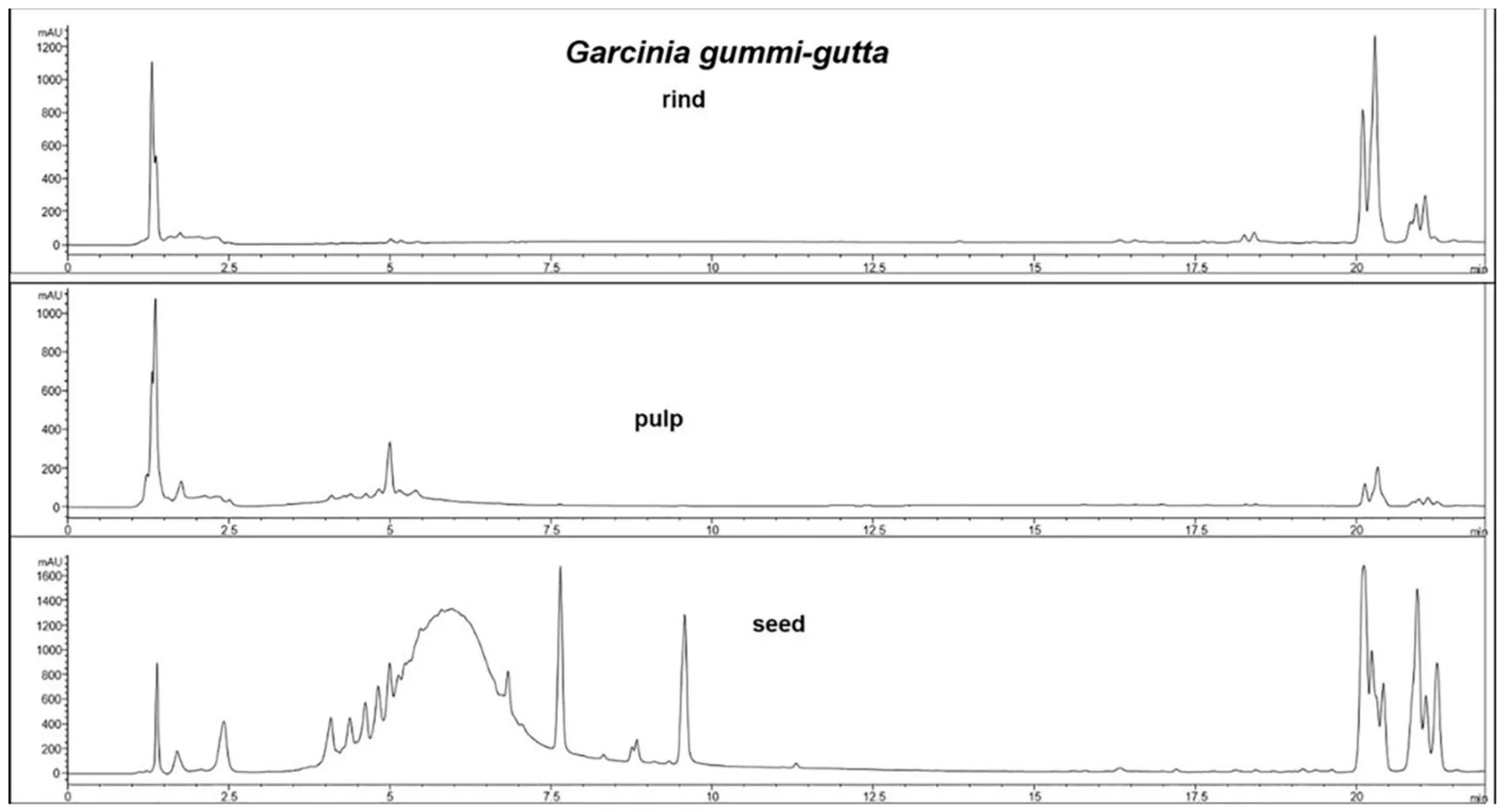
Liquid Chromatography–Mass Spectrometry chromatograms for *G. gummi-gutta* rind, pulp and seed.

The main compounds found in *G. gummi-gutta* freeze-dried rind were hydroxycitric acid (28.8% *w*/*w*) and citric acid (3.76% *w*/*w*) (Table 3). The concentrations of hydroxycitric acid and citric acid in the freeze-dried pulp were 9.55% *w*/*w* and 5.74% *w*/*w*, respectively (Table 4). The rind, pulp and seed contained tocotrienic acid aldehyde and guttiferone derivative (garcinol). As with *G. dulcis*, the seed contained benzylamine derivatives, which indicate the presence of amine/amino acids, and the biflavone, morelloflavone (Table 5). LC-MS chromatograms for *G. gummi-gutta* rind, pulp and seed are presented in Figure 6. Further details of the LC-MS chromatogram and UV–visible spectra can be found in Supplementary Figures S10–S12.

Further information on LC-MS analyses is presented in Supplementary Figures S14–S24.

## 4. Discussion

*Garcinia* species are distributed mainly in tropical regions, including the areas of northern Australia [7,34]. Exotic fruits, including *G. mangostana*, were originally introduced to Australia over 100 years ago in the late 1800s [35] and then naturalised [7]. Within Australia, *G. mangostana* is primarily planted in northern Queensland and Northern Territory as a naturalised species [7,36]. *G. dulcis* is usually restricted to northern Queensland, where it grows in rainforest from the Melville Range, Cape York Peninsula to the Torres Strait islands [7], but it is also grown around southeast Queensland [37]. *G. dulcis* is widely distributed in Southeast Asia, India and New Guinea [7,23]. *G. humilis* was introduced from Bolivia and grows well in the Burdekin Shire, north Queensland [10]. *G. gummi-gutta* is native to Southeast Asia [38] and most likely was introduced as backyard fruit to Australia.

*Garcinia* fruits are recognised as sources of bioactive compounds including organic acids (citric acid, hydroxycitric acid), prenylated benzophenones, xanthones, triterpenes and flavonoids [1,16,39]. Benzophenones are also present in other plants and fungi, but they are mainly found in the Clusiaceae family [40]. Xanthones from higher plants are mainly associated with the families of Clusiaceae and Gentianaceae [41]. Our analysis showed the presence of complex phytochemicals in the rind, pulp and seeds of *G. mangostana*, *G. humilis*, *G. dulcis*, and *G. gummi-gutta*, which varied between species, including organic acids (hydroxycitric acid and citric acid), phenolics, anthocyanins, flavanols, procyanidins, flavone glycosides, bi-flavanone glycosides, xanthones and their derivatives, and depsidones. Overall, there were higher concentrations of phytochemicals in the rind and seed than in the pulp, as reported previously [42,43].

In traditional medicine, *G. mangostana* fruits and parts of the plants have been used to treat many health issues including inflammation, microbial infection, obesity and cancer [15,44]. *G. dulcis*, native to Southeast Asia, may produce similar responses [45,46,47,48]. Further, hydroxycitric acid from *G. gummi-gutta* may reduce obesity [38,49]. In addition, we have reported that *G. humilis*, *G. dulcis* and *G. mangostana* show cardioprotective, hepatoprotective, anti-obesity and anti-inflammatory properties and promote a healthier gut microbiome in a rat model of diet-induced metabolic syndrome [12,24,25]. Other *Garcinia* species such as *G. indica* (kokum) found in India produced anti-obesity, anti-diabetic, cardioprotective, and chemopreventive responses [50].

As reported previously, *G. gummi-gutta* (previously *G. cambogia*) rind and pulp samples demonstrated the presence of hydroxycitric acid [51,52]. We measured around 29% *w*/*w* of hydroxycitric acid in the freeze-dried rind, higher than reported in India (16–18% *w*/*w*). However, we used freeze-dried samples compared to fresh fruit used in the previous study [52]. Extracted samples provided about 42–44% *w*/*w* dw of hydroxycitric acid [53]. However, in comparison to *G. gummi-gutta*, *G. indica* and *G. atroviridis* from Asia, which mostly contain hydroxycitric acid [54], we detected very low amounts of hydroxycitric acid in *G. humilis* rind and pulp samples and none in *G. dulcis* rind and pulp samples, although a recent report showed 32% *w*/*w* hydroxycitric acid in the rind and seed of *G. dulcis* [55]. Instead, we found substantial amounts of citric acid in *G. humilis* rind and pulp, *G. gummi-gutta* fruits, and *G. dulcis* pulp and rind. The concentration of citric acid in rind and pulp of *G. dulcis* (19–24% *w*/*w* dw) was higher than the amount present in lemons and limes (8% *w*/*w*) [56]. Citric acid is used widely in topical formulations for skin health and shows antioxidant and anti-inflammatory activities [57,58].

Flavonoids have a C_6_-C_3_-C_6_ structure, and the subgroups of dietary significance are anthocyanins, flavanols, flavones, isoflavones, flavan-3-ols, and flavanones, while minor flavonoids include dihydroflavanols, flavan-3,4-diols, coumarins, chalcones, dihydrochalcones, and aurones [56]. Combinations of polyphenols (flavones and flavanols) have synergistic properties [59,60]. Luteolin and kaempferol, for instance, showed synergistic properties against herpes simplex virus [59] and decreased claudin-2 expression and proliferation in A549 cells [61]. The combination of quercetin, kaempferol and pterostilbene at low concentrations synergistically reduced intracellular reactive oxygen species [62]. Furthermore, a formulation of quercetin and kaempferol showed superior anti-ageing activity in a D-galactose-induced skin ageing mouse model [63]. Our studies have proposed that the flavonoids in *Garcinia* fruit components may act synergistically with other phytochemicals such as citric acid found together in the fruit, to produce the observed health-promoting effects [12,24].

Morelloflavone is a biflavonoid with two covalently linked flavones, apigenin and luteolin [64]. In our analysis, morelloflavone was present in *G. dulcis* rind, pulp, and seed, which is consistent with previous reports [23,47,65]. Morelloflavone inhibited the cholesterol-synthesising enzyme HMG-CoA reductase activity in vitro [66] and prevented atherosclerosis in mice by reducing vascular smooth muscle cell migration into the intima layer [67]. Morelloflavone also inhibited the human secretory phospholipase A_2_ and has anti-inflammatory activities [68]. Additionally, *G. dulcis* flower extract containing camboginol and morelloflavone induced autophagic cell death in human glioblastoma cells [69].

Proanthocyanidins, also known as condensed tannins or catechin tannins, are the most abundant plant-derived polyphenols, most commonly found in berries and fruits [70,71]. Procyanidins are the most common proanthocyanidins, and their monomers have potential beneficial effects on human health, including immunomodulatory, anti-inflammatory, anti-cancer, antioxidant, cardioprotective and anti-thrombotic properties [72]. In our analysis, we describe the presence of B-type and A-type procyanidins in the rind and pulp of *G. humilis* and in the rind and seeds of *G. mangostana*. The presence of procyanidins in *G. humilis* rind was not previously reported; we have recently suggested that the cardioprotective effects of *G. humilis* are due to the procyanidins [12].

More than 300 naturally occurring benzophenones have been isolated and identified, most of them in higher plants, with around half as polyprenylated benzophenones and the other half with basic benzophenone skeletons [40]. Of these compounds, most originated from the Clusiaceae family [40,73]. Polyprenylated benzophenones can be categorised into four types: type-A, with the complete benzoyl group linked at C-1; type-B, with the benzoyl group linked at C-3; type-C, with the benzoyl group at C-5; and type-D, without the eight-membered ring and with a modified conjugated system in the B-ring formed and saturated due to the addition of carbonyl groups [40]. Type-B polyprenylated benzophenones are the most common type of naturally occurring benzophenones and are the most abundant polyprenylated benzophenones isolated from the *Garcinia* genus [40]. In *G. humilis*, guttiferone A and guttiferone I have been reported [74,75], but we did not detect these compounds in our analysis. Instead, we detected the presence of garcinol, another type-B benzophenone. As guttiferone A and garcinol have similar molecular mass and structure, and possibly share the same UV–visible spectra, it is very difficult to differentiate these compounds without using a verified standard. Garcinol and its derivative, isogarcinol, were also present in the rind and pulp of *G. dulcis* and *G. gummi-gutta*. Garcinol has beneficial effects against chronic diseases such as diabetes, cardiovascular disease, cancer, neurodegenerative disease, and allergy, and also has anti-microbial, anti-inflammatory, and antioxidant properties [76,77]. Garcinol is one of the most abundant polyisoprenylated benzophenones from *Garcinia* plants [17].

Xanthones are a class of polyphenols with the skeleton xanthene-9-one [15]. The presence of α-mangostin, β-mangostin, and γ-mangostin in *G. mangostana* rind and pulp samples was similar to previous studies on this fruit [15,78,79,80]. We detected 5.42% *w*/*w* of α-mangostin in the freeze-dried rind of *G. mangostana* compared to a previous report showing about 1.82–2.47% *w*/*w* α-mangostin from Thailand [81]. The climate, rainfall, temperature, and geographical locations altered the α-mangostin content in the rind [82]. In our analysis, we found a very small amount of prenylated xanthones in *G. humilis*, which is consistent with other findings [83]. The presence of various xanthones, including mangostanol and γ-mangostin, in *G. humilis* peel was recently reported [84]. Xanthone derivatives are most prevalent in *G. mangostana* seeds, while the *G. dulcis* seed sample has mostly xanthenone derivatives. Xanthones from *Garcinia* species, particularly α-mangostin, have beneficial health properties, including cardioprotective, anti-inflammatory, antioxidant, anti-obesity, analgesic and anti-cancer responses [21,85,86]. The discovery of xanthones in *G. humilis* could suggest its potential as a new source of this phytochemical. Overall, a wide variety of compounds such as xanthones and benzophenones can be produced by different species of *Garcinia* plants. However, due to the limitations in our methods, we could not fully characterise the benzophenones present.

Previous characterisation of anthocyanins in *G. mangostana* showed the presence of cyanidin 3-sophoroside and cyanidin 3-glucoside [87]. Our results agree with previous findings showing the presence of anthocyanins in the rind but not the pulp [42]. Among the flavonoid C-glycosides, flavone C-glycosides such as vitexin, isoorientin, orientin, isovitexin and their multiglycosides are frequently mentioned [88]. In this study, we discovered the presence of isovitexin glycosides and depsidones in *G. humilis* rind, which has not been reported previously. Seeds of both *G. gummi-gutta* and *G. dulcis* contain biflavone compounds and their derivatives. Furthermore, we found tocotrienoic acid derivatives from the vitamin E family of compounds in the rind of *G. gummi-gutta*, the pulp of *G. gummi-gutta*, and *G. mangostana*, and in the seeds of all fruits except *G. humilis*. The presence of tocotrienols in all parts of *G. gummi-gutta* fruits and *G. dulcis* has not been reported previously. Overall, the analysis of the seed parts showed a complex mixture of phytochemicals including chlorogenic and coumaric acids.

In this study, we analysed four species of *Garcinia* fruits, which are only a sample of the *Garcinia* fruits grown in Australia, including the native Australian *Garcinia* fruits [7]. Further, samples of fruit from different species from different locations were not available. In addition, some compounds were identified by comparing the UV–visible spectra to known compounds; hence, we may not be able to separate closely related compounds. Thus, more detailed analyses are required to increase current knowledge. Comparing HPLC retention times to standards is an additional step in identification, but additional methods such as Nuclear Magnetic Resonance spectroscopy, Mass Spectrometry and crystallography are suggested [89].

## 5. Conclusions

The chemical analysis of *G. mangostana*, *G. gummi-gutta*, *G. dulcis* and *G. humilis* revealed the presence of many phytochemicals that are ubiquitously found in nature. We confirmed the presence of xanthones characteristic of *Garcinia* species in *G. mangostana*, with α-mangostin as the major xanthone in the rind. Garcinol was the main phytochemical in the rind of *G. dulcis*. In agreement with previous studies, we discovered hydroxycitric acid to be the main organic acid in *G. gummi-gutta* but not in *G. humilis*. Citric acid was predominant in *G. humilis*, *G. dulcis* and *G. gummi-gutta*. The analysis of *G*. *humilis* revealed the presence of many compounds such as procyanidins, benzophenones and citric acid. Overall, we discovered that there are more bioactive compounds and greater concentrations in the rind and seed than in the pulp. We found that the phytochemicals in the rind of *G. mangostana* and *G. humilis* are more complex. This discovery provides additional knowledge and suggests the extraction of beneficial phytochemicals from the rind and seed, which are usually regarded as food waste. In addition, phytochemical discovery from these *Garcinia* fruits further supports their use as potential sources of pharmaceuticals and nutraceuticals.

## Figures and Tables

**Figure 2:**
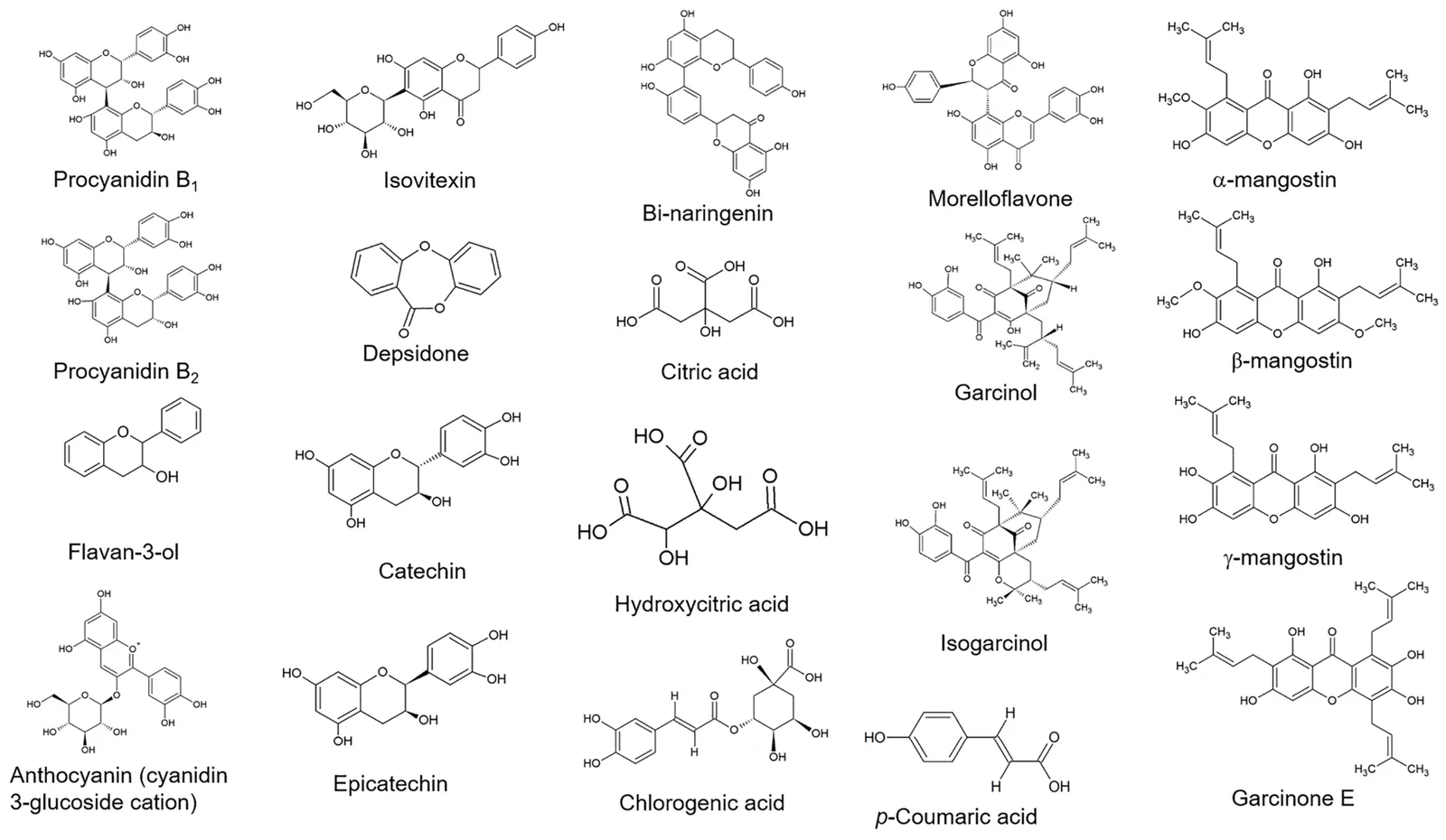
The structures of phytochemicals found in tropical Australian *Garcinia* species (*G. humilis*, *G. mangostana*, *G. dulcis* and *G. gummi-gutta*).

**Table 1:** Calibration curve reference standard concentration ranges and detection wavelength.

Phytochemical	Concentration Range	Detection Wavelength
Flavanols (catechin, epicatechin)	0.001002–1.002 mg/mL	280 nm
Procyanidin B_2_	0.0032–0.236 mg/mL	280 nm
Procyanidin A_2_	0.0013–0.065 mg/mL	280 nm
Hydroxy citric acid and lactone	0.02225–0.89 mg/mL	215 nm
Quinic acid	0.109–0.545 mg/mL	210 nm
Malic acid	0.164–0.82 mg/mL	210 nm
Citric acid	0.0177–0.71 mg/mL	215 nm
Anthocyanins	calculated based on single peak area of cyanidin 3-glucoside chloride	535 nm
Rutin	0.0118–0.59 mg/mL	254 nm
Quercetin	0.0028–0.142 mg/mL	254 nm
α-Mangostin	0.00824–0.412 mg/mL	240 nm
Garcinol	0.005656–0.2828 mg/mL	240 nm
Naringin	0.0086–0.43 mg/mL	280 nm
Chlorogenic acid	0.0398–0.398 mg/mL	330 nm

**Table 2:** Total phenolic contents of rind, pulp and seeds of *G. humilis*, *G. mangostana*, *G. dulcis* and *G. gummi-gutta*.

Species	Rind	Pulp	Seed
*G. humilis*	147.33 ± 0.55 ^a,B^	40.93 ± 0.21 ^c,D^	66.72 ± 0.12 ^b,D^
*G. gummi-gutta*	36.66 ± 0.05 ^c,D^	102.24 ± 0.05 ^b,A^	214.32 ± 0.05 ^a,A^
*G. dulcis*	103.79 ± 0.18 ^b,C^	77.28 ± 0.12 ^c,B^	127.36 ± 0.05 ^a,C^
*G. mangostana*	222.08 ± 0.05 ^a,A^	60.58 ± 0.05 ^c,C^	173.92 ± 0.05 ^b,B^

Total phenolic content was expressed as mg gallic acid equivalent/g dry weight. Different lowercase superscript indicates significant difference between columns (different parts of the same species compared). Different uppercase superscript indicates significant difference between rows (same fruit part of different species compared). *p* < 0.05, assessed by two-way ANOVA.

**Table 3:** The compounds identified (LC-MS analysis) and quantified (HPLC analysis) in the rind of *G. humilis*, *G. mangostana*, *G. dulcis* and *G. gummi-gutta*.

Compound		Molecular Weight	Absorbance Maxima (nm), λ_(max)_	Fragment Ions (M+H)^+^, *m*/*z*	*G. humilis* (% *w*/*w* dw)	*G. mangostana* (% *w*/*w* dw)	*G. dulcis* (% *w*/*w* dw)	*G. gummi-gutta* (% *w*/*w* dw)
Citric acid		192	NA	193	12.14	ND	24	3.76
Hydroxycitric acid		208	NA	209	Detected	0.42	ND	28.83
Phenolic acid (coumaric acid)		118, 146	230, 315	119, 147	ND	ND	Detected	ND
Anthocyanins		207	280, 520	208	ND	0.14	ND	ND
Anthocyanins, amino acids, amines		137, 215, 233, 247, 261, 275	NA	136, 216, 234, 248, 262, 276	ND	Detected	ND	ND
Flavan-3-ol	Catechin	290	280	291	0.099	ND	ND	ND
	Epicatechin	290	280	291	1.005	Detected	ND	ND
Flavanol derivatives (procyanidins)		246, 290, 368, 562	290	247, 291, 369, 563	Detected	Detected	ND	ND
Procyanidins	Procyanidin B_1_	578	280	579	Detected	Detected	ND	ND
	B-type procyanidin, dimer (procyanidin B_2_)	578	280	579, 291	Detected 4.898	Detected	ND	ND
	Procyanidin A_2_	578	280	579	0.043	ND	ND	ND
Flavone C-glycosides	Isovitexin	432	220, 270, 350	313, 433, 579	Detected	ND	ND	ND
	Glycoside (rhamnose)	578						
Flavanone derivative		Supplementary Figure S13	290	Supplementary Figure S13	Detected	ND	ND	ND
Tetrahydroxy-prenyl-xanthones isomer		272, 328	250, 285, 330	273, 329	Detected	ND	ND	ND
Hydroxy-dimethoxy-prenyl-xanthones		272, 284, 340	255, 325	273, 285, 341	Detected	ND	ND	ND
Tetrahydroxy-diprenyl-xanthone		272, 396	250, 285, 350	273, 397	Detected	ND	ND	ND
Geranyl-tetrahydroxy-prenyl-xanthone isomer		284, 340, 408, 464	250, 330	285, 341, 409, 465	Detected	ND	ND	ND
Total xanthones *		-	-	-	0.0423	6.494	ND	ND
Xanthone derivatives (γ-mangostin) or gartanin, 396		340, 396	205, 245, 260, 320, 365	341, 397	ND	Detected	ND	ND
Xanthone derivatives (α-mangostin)		410	200, 245, 260, 320, 365	355, 411	ND	5.416	ND	ND
Xanthone derivatives (β-mangostin)		424	205, 245, 260, 315	369, 425	ND	Detected	ND	ND
Xanthone derivatives (garcinone E)		464	245, 260, 320, 370	465	ND	Detected	ND	ND
Depsidones		412, 426	270–280	413, 427	Detected	ND	ND	ND
Tocotrienoic acid aldehyde		410	294–296	411	ND	ND	ND	Detected
Biflavone (trihydroxyflavone-trihydroxyflavone)		540	290, 330	541	ND	ND	Detected	ND
Bi-flavanone glycoside	Bi-naringenin	542	230, 290	543, 705	Detected	ND	ND	ND
	Glycoside (glucose)	704						
Bi-flavanone (Bi-naringenin)		542	230, 290	543	Detected	ND	ND	ND
Biflavone glycoside (morelloflavone) (tetrahydroxyflavone-trihydroxyflavone-glucoside)		556, 718	210, 290, 345	557, 719	ND	ND	Detected	ND
Biflavone (morelloflavone) (tetrahydroxyflavone-trihydroxyflavone)		556	210, 290, 345	557	ND	ND	Detected	ND
biflavone (Morelloflavone derivative) (tetrahydroxyflavone-tetrahydroxyflavone) derivative		556	210, 290, 345	557	ND	ND	Detected	ND
Garcinol		603	200, 245, 350	604	ND	ND	6.348	Detected
Benzophenone derivative		Tentative ID based on common UV–visible spectra	245, 370	Tentative ID based on common UV–visible spectra	ND	ND	Detected	ND
Total benzophenones ^#^		Tentative ID based on common UV–visible spectra	250, 280, 330	Tentative ID based on common UV–visible spectra	ND	ND	7.863	Detected

ND = not detected. * Total xanthones was calculated as α-mangostin. ^#^ Total benzophenones was calculated as garcinol.

**Table 4:** The compounds identified (LC-MS analysis) and quantified (HPLC analysis) in the pulp of *G. humilis*, *G. mangostana*, *G. dulcis* and *G. gummi-gutta*.

Compound		Molecular Weight	Absorbance Maxima (nm), λ_(max)_	Fragment Ions (M+H)^+^, *m*/*z*	*G. humilis* (% *w*/*w* dw)	*G. mangostana* (% *w*/*w* dw)	*G. dulcis* (% *w*/*w* dw)	*G. gummi-gutta* (% *w*/*w* dw)
Citric acid		192	NA	193	1.44	ND	19	5.74
Hydroxycitric acid		208	NA	209	Detected	0.17	ND	9.55
Phenolic acid (coumaric acid)		118, 146	230, 315	119, 147	ND	ND	Detected	ND
Anthocyanins		207	280, 520	208	ND	ND	ND	ND
Flavan-3-ol	Catechin	290	280	291	0.015	ND	ND	ND
	Epicatechin	290			0.032			
Procyanidin (B-type procyanidin, dimer—procyanidin B_2_)		578	280	579, 291	0.32	ND	ND	ND
Flavone C-glycoside	Isovitexin	432	265, 350	313, 433, 579	Detected	ND	ND	ND
	Glycoside (rhamnose)	578						
Flavanone derivative		Supplementary Figure S13	290	Supplementary Figure S13	Detected	ND	ND	ND
Geranyl-tetrahydroxy-prenyl-xanthone isomer		284, 340, 408, 464	250, 330	285, 341, 409, 465	Detected	ND	ND	ND
Trihydroxy-prenyl xanthone		326	240, 265, 310, 380	327	ND	Detected	ND	ND
Xanthone derivatives (γ- mangostin,)		340, 396	205, 245, 260, 320, 365	341, 397	ND	Detected	ND	ND
Xanthone derivatives (α-mangostin,)		410	205, 240, 260, 320, 370	355, 411	ND	0.0867	ND	ND
Tetrahydroxy-geranyl-prenyl-xanthone (garcinone E)		464	245, 260, 320, 370	465	ND	Detected	ND	ND
Dihydroxy-prenyl-xanthone-carboxylic acid		408	290, 330	409	ND	Detected	ND	ND
Dihydroxy-dimethyl-trimethyl-tridecatrienyl-benzopyranone (parvifoliol E)		410	280	411	ND	Detected	ND	ND
Dihydroxy-dimethyl-trimethyl-tridecatrienyl-benzopyranone (parvifoliol F)		396	300	397	ND	Detected	ND	ND
Total xanthones *		-	-	-	ND	0.173	ND	ND
Tocotrienoic acid aldehyde		410	294–296	411	ND	Detected	ND	Detected
Biflavone (trihydroxyflavone-trihydroxyflavone)		540	290, 330	541	ND	ND	Detected	ND
Biflavone glycoside (morelloflavone)(tetrahydroxyflavone-trihydroxyflavone-glucoside)		556, 718	210, 290, 345	557, 719	ND	ND	Detected	ND
Biflavone (morelloflavone) (tetrahydroxyflavone-trihydroxyflavone)		556	210, 290, 345	557	ND	ND	Detected	ND
Biflavone (morelloflavone derivative) (tetrahydroxyflavone-tetrahydroxyflavone) derivative		556	210, 290, 345	557	ND	ND	Detected	ND
Guttiferone derivative (isogarcinol)		342, 398, 410, 466, 478, 602	230, 275	343, 399, 411, 467, 479, 603	ND	ND	Detected	ND
Guttiferone derivative (garcinol)		602	200, 245, 350	603	ND	ND	2.874	Detected
Benzophenone derivative		Tentative ID based on common UV–visible spectra	245, 370	Tentative ID based on common UV–visible spectra	ND	ND	Detected	ND
Total benzophenones ^#^		-	-	-	ND	ND	3.539	Detected

ND = not detected. * Total xanthones was calculated as α-mangostin. ^#^ Total benzophenones was calculated as garcinol.

**Table 5:** The compounds identified (LC-MS analysis) and quantified (HPLC analysis) in the seeds of *G. humilis*, *G. mangostana*, *G. dulcis* and *G. gummi-gutta*.

Compound	Molecular Weight	Absorbance Maxima (nm), λ_(max)_	Fragment Ions (M+H)^+^, *m*/*z*	*G. humilis* (% *w*/*w* dw)	*G. mangostana* (% *w*/*w* dw)	*G. dulcis* (% *w*/*w* dw)	*G. gummi-gutta* (% *w*/*w* dw)
Amine/alkaloid (benzylamine derivative)	143	200, 260, 300	144, 162	ND	ND	Detected	Detected
Caffeoyl-quinic acid (chlorogenic acid)	162, 354	220, 330	163, 355	ND	Detected	Detected	ND
Phenolic acid (coumaric acid)	118, 146	230, 315	119, 147	ND	ND	Detected	ND
Phenolic compounds	236	200, 280	237	ND	Detected	ND	ND
Flavanols (catechin, procyanidin)	290, 578	205, 280	291, 579	ND	Detected	ND	Detected
Flavone C-glycoside	312, 432	265, 350	313, 433	ND	Detected	ND	ND
Trihydroxy-prenyl xanthone	326	240, 265, 310, 380	327	ND	Detected	ND	ND
Trihydroxy-triprenyl-xanthone	380	240, 265, 315, 380	381	ND	Detected	ND	ND
Dihydroxy-dimethyl-trimethyl-tridecatrienyl-benzopyranone (parvifoliol F)	396	200, 300	397	ND	Detected	ND	ND
Dihydroxy-prenyl-xanthone-carboxylic acid	408	200, 290, 330	409	ND	Detected	ND	ND
Tocotrienoic acid aldehyde	410	294–296	411	ND	Detected	Detected	Detected
Trihydro-methoxy-diprenyl-xanthone (α-mangostin)	410	200, 245, 260, 320, 365	411	ND	Detected	ND	ND
Dihydroxy-dimethyl- trimethyl-tridecatrienyl-benzopyranone (parvifoliol E)	410	200, 275	411	ND	Detected	ND	ND
Tetrahydroxy-geranyl-prenyl-xanthone (garcinone E)	464	245, 260, 320, 370	465	ND	Detected	ND	ND
Diprenyl-xanthenone derivative	466, 484	210, 340	467, 485	ND	ND	Detected	ND
Diprenyl-xanthenone derivative	480, 498	205, 340	481, 499	ND	ND	Detected	ND
Diprenyl-xanthenone derivative	484, 516	200, 230, 275, 345	485, 517	ND	ND	Detected	ND
Diprenyl-xanthenone derivative	498, 516	200, 230, 275, 335	499, 517	ND	ND	Detected	ND
Biflavone (trihydroxyflavone-trihydroxyflavone)	540	200, 215, 290	541	ND	ND	Detected	Detected
Biflavone glycoside (trihydroxyflavone-trihydroxyflavone-glucoside)	540, 702	200, 290, 330	541, 703	ND	ND	ND	Detected
Biflavone glycoside (morelloflavone glucoside) (tetrahydroxyflavone-trihydroxyflavone -glucoside)	556, 718	205, 275, 290, 345	557, 719	ND	ND	Detected	Detected
Biflavone (morelloflavone) (tetrahydroxyflavone-trihydroxyflavone)	556	215, 290, 345	557	ND	ND	Detected	Detected
Biflavone (morelloflavone derivative) (tetrahydroxyflavone-tetrahydroxyflavone) derivative	556	200, 290, 345	557	ND	ND	Detected Total morelloflavone (2.9)	ND
Di-deoxy-guttiferone derivative	570	200, 245, 310	571	ND	ND	ND	Detected
Deoxy-guttiferone derivative	586	205, 245, 300	587	ND	ND	ND	Detected
Guttiferone derivative	342, 398, 410, 466, 478, 534	240, 260- 270	343, 399, 411, 467, 479, 535	Detected	ND	ND	ND
Guttiferone derivative	326, 394, 450, 518, 586, 600	250–260	327, 395, 451, 519, 587, 601	Detected	ND	ND	ND
Guttiferone derivative (isogarcinol)	342, 398, 410, 466, 478, 602	230, 275	343, 399, 411, 467, 479, 603	Detected	ND	ND	ND
Guttiferone derivative (garcinol)	342, 398, 411, 467, 479, 603	200, 245, 350	343, 399, 411, 467, 479, 603	2.691	ND	ND	Detected
Total benzophenones ^#^	-	-	-	3.566	ND	ND	ND

ND = not detected. ^#^ Total benzophenone was calculated as garcinol.

## Data Availability

The original contributions presented in this study are included in the article/Supplementary Materials. Further inquiries can be directed to the corresponding author.

## Supplementary Materials

The following supporting information can be downloaded at https://www.mdpi.com/article/10.3390/foods15203720/s1. Figure S1: LC-MS chromatogram and UV–visible spectra of *G. mangostana* rind; Figure S2: LC-MS chromatogram and UV–visible spectra of *G. mangostana* pulp; Figure S3: LC-MS chromatogram and UV–visible spectra of *G. mangostana* seeds; Figure S4: LC-MS chromatogram and UV–visible spectra of *G. humilis* rind; Figure S5: LC-MS chromatogram and UV–visible spectra of *G. humilis* pulp; Figure S6: LC-MS chromatogram and UV–visible spectra of *G. humilis* seeds; Figure S7: LC-MS chromatogram and UV–visible spectra of *G. dulcis* rind; Figure S8: LC-MS chromatogram and UV–visible spectra of *G. dulcis* pulp; Figure S9: LC-MS chromatogram and UV–visible spectra of *G. dulcis* seeds; Figure S10: LC-MS chromatogram and UV–visible spectra of *G. gummi-gutta* rind; Figure S11: LC-MS chromatogram and UV–visible spectra of *G. gummi-gutta* seeds; Figure S12: LC-MS chromatogram and UV–visible spectra of *G. gummi-gutta* seeds; Figure S13: *G. humilis* peak #6; Figure S14: *G. humilis* organic acid chromatogram; Figure S15: *G. humilis* procyanidin chromatogram; Figure S16: *G. dulcis* chromatogram–citric acid overlays; Figure S17: *G. gummi-gutta* chromatogram – organic acid overlays; Figure S18: Calibration curves: procyanidins; Figure S19: Calibration curves: organic acids (quinic acid, citric acid, malic acid); Figure S20: Calibration curves: hydroxycitric acid; Figure S21: Calibration curves: flavonoids (rutin and quercetin); Figure S22: Calibration curves: garcinol and xanthone (α-mangostin); Figure S23: Calibration curves: naringin; Figure S24: Calibration curves: phenolic acids (chlorogenic acid).
