# Supplementary material for: Phytochemical Constituents of Selected Garcinia Fruits from Tropical Australia
Source: Foods. 2026 Oct 19;15(20):3720. doi: 10.3390/foods15203720 (PMC13704158; doi:10.3390/foods15203720)
Supplement: Supplementary file 1 [file foods-15-03720-s001.zip › foods-4583695-supplementary.pdf]

foods

MDPI

Article

Phytochemical Constituents of Selected *Garcinia* Fruits from Tropical Australia

Oliver D. John [1,†], Sunil K. Panchal [1,‡], Lindsay Brown [1,§], Peter Mouatt [2,*]

1 Functional Foods Research Group, University of Southern Queensland, Toowoomba, QLD 4350, Australia; odjohn@ums.edu.my (O.D.J.); s.panchal@westernsydney.edu.au (S.K.P.); lindsay.brown@griffith.edu.au (L.B.)
2 Analytical Research Laboratory, Southern Cross Plant Science, Southern Cross University, East Lismore, NSW 2480, Australia
* Correspondence: peter.mouatt@scu.edu.au (P.M.); Tel. +61-2-6622-3211
† Current address: Faculty of Food Science and Nutrition, Universiti Malaysia Sabah, Kota Kinabalu 88400, Sabah, Malaysia.
‡ Current address: School of Science, Faculty of Engineering, Computing and Science, Western Sydney University, Hawkesbury Campus, Richmond, NSW 2753, Australia.
§ Current address: School of Pharmacy and Medical Science, Griffith University, Southport, QLD 4222, Australia.

Supplementary Material

https://doi.org/10.3390/foods15203720

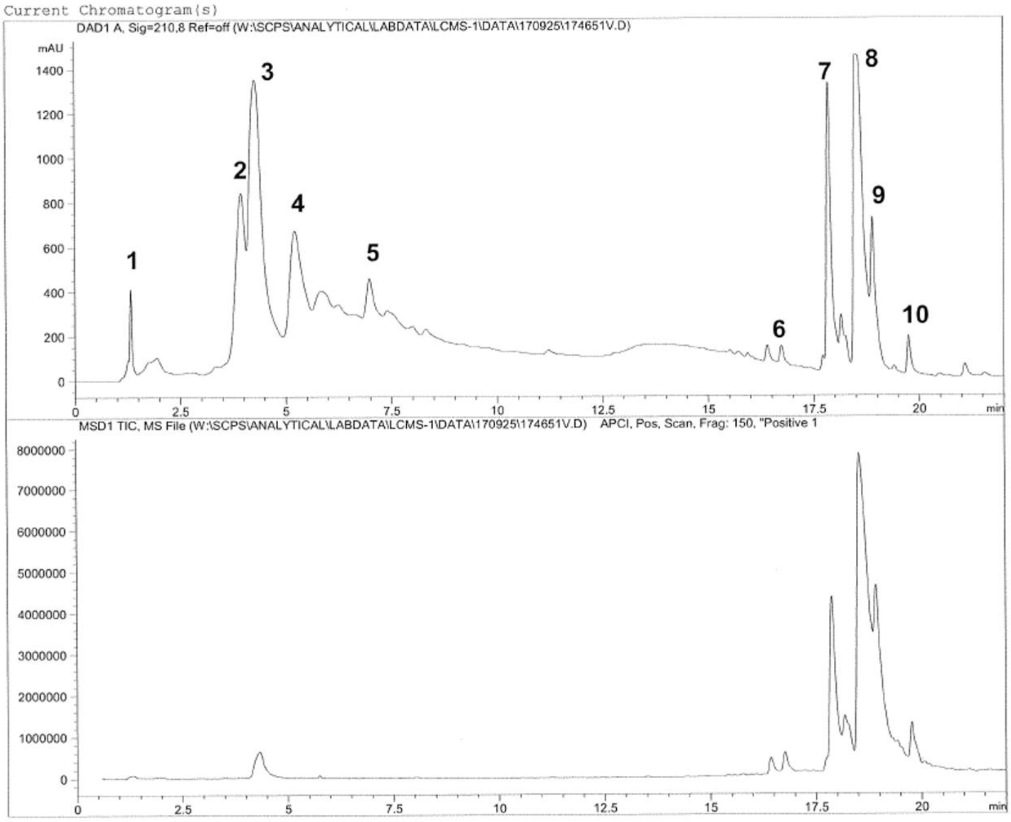

Peak	Fragment ions [M + HI]	UV	Tentative ID (MW)
1	136, 216, 234, 248, 262, 276	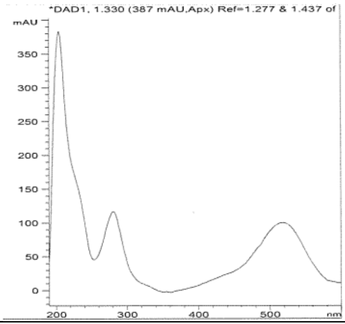	Mixed peak (anthocyanins, amino acids, amines)
2	579	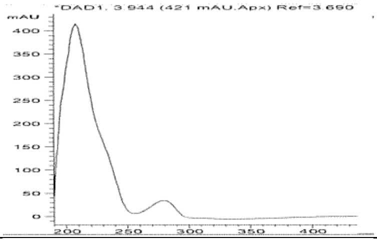	Procyanidin (procyanidin B1)
3	291	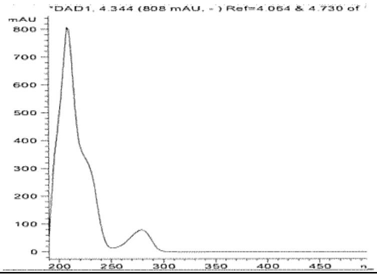	Flavan-3-ol (epicatechin)

4	247, 291, 369, 563	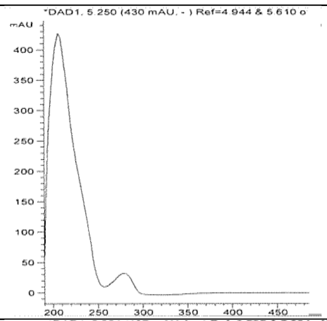	Flavanol derivative (procyanidin)
5	247, 369, 563	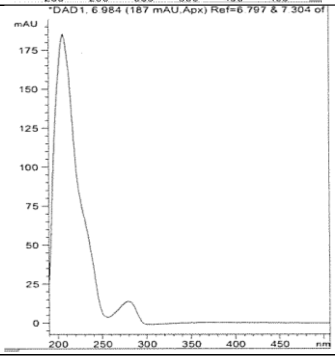	Flavanol derivative (procyanidin)
6	341, 397, 355, 411	-	Xanthone derivatives (mixture of xanthones)
7	341, 397	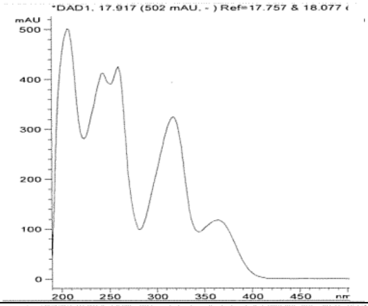	Xanthone derivative (γ-mangostin)
8	355, 411	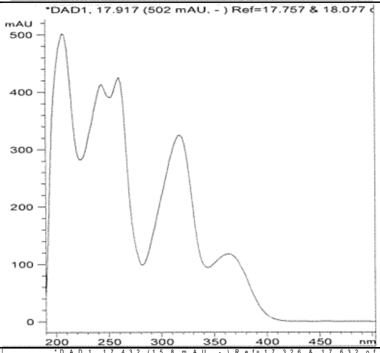	Xanthone derivative (α-mangostin)
9	465	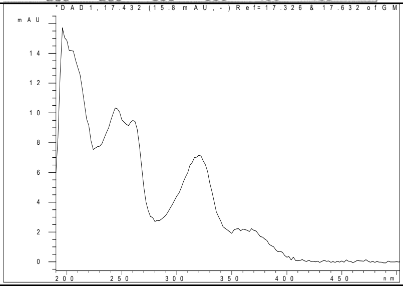	Tetrahydroxy-geranyl-prenyl-xanthone (garcinone E)

10	369, 425	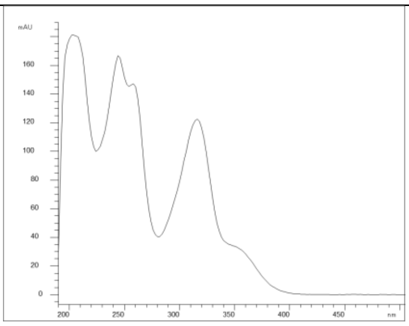	Xanthone derivative (β-mangostin)

Figure S1. LC-MS chromatogram and UV–visible spectra of *G. mangostana* rind.

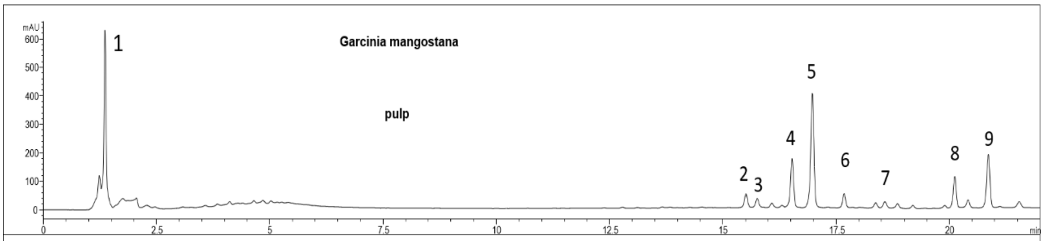

Peak	Fragment ions [M + HI]	UV	Tentative ID (MW)
1	136, 216, 234, 248, 262, 276	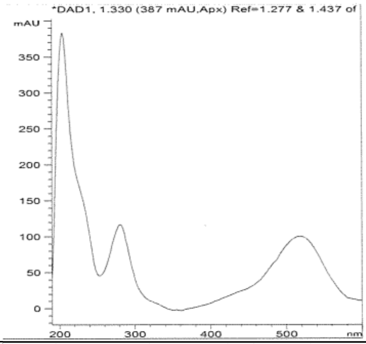	Mixed peak (anthocyanins, amino acids, amines)
2	327	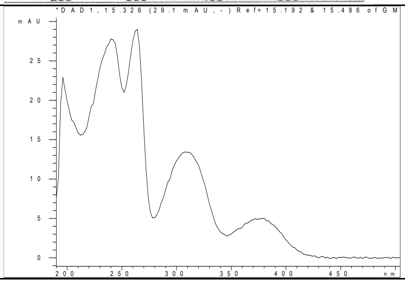	Trihydroxy-prenyl-xanthone
3	341, 397	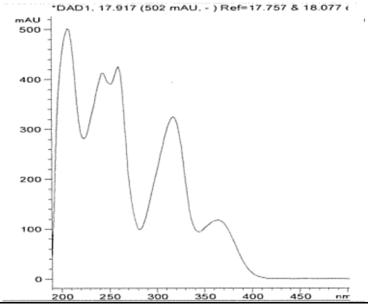	Xanthone derivative (γ-mangostin)

4	327	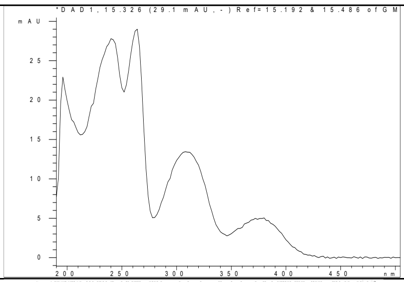	Trihydroxy-prenyl-xanthone
5	355, 411	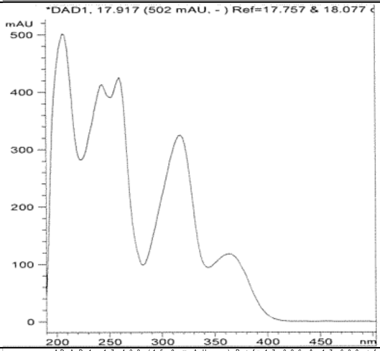	Xanthone derivative (α-mangostin)
6	465	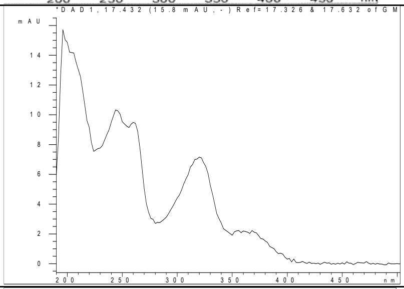	Tetrahydroxy-geranyl-prenyl-xanthone (Garcinone E)
7	409	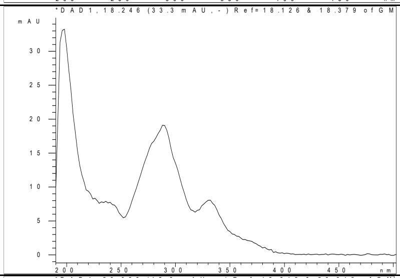	Dihydroxy-prenyl-xanthone-carboxylic acid
8	411	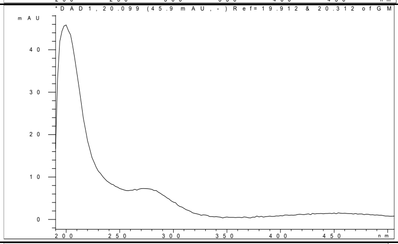	Dihydroxy-dimethyl- trimethyl-tridecatrienyl-benzopyranone (parvifoliol E)
9	397	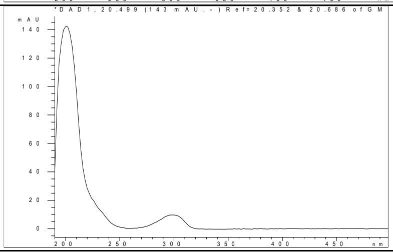	Dihydroxy-dimethyl- trimethyl-tridecatrienyl-benzopyranone (parvifoliol F)

Figure S2. LC-MS chromatogram and UV–visible spectra of *G. mangostana* pulp.

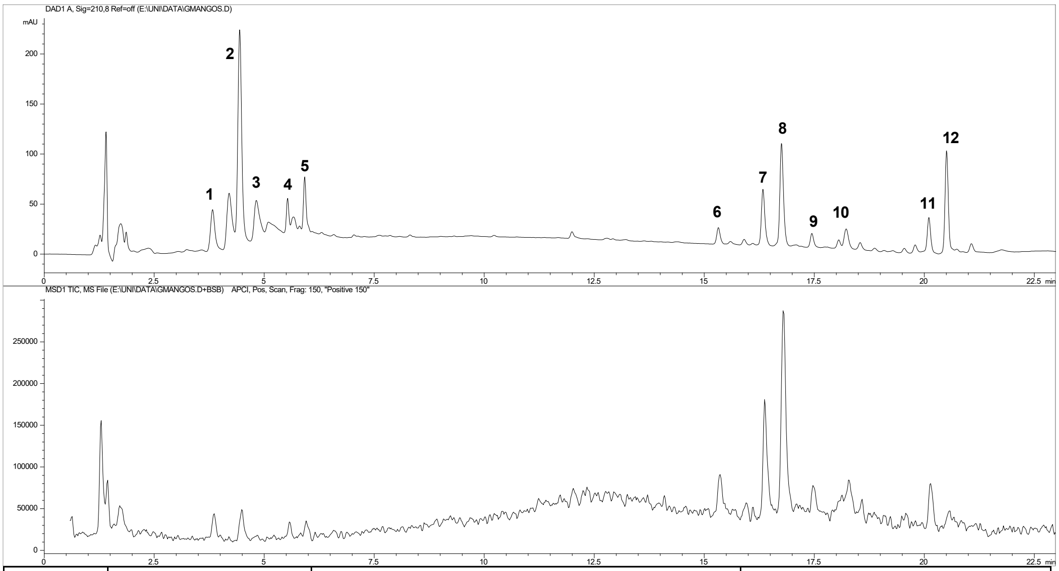

Peak	Fragment ions [M + HI]	UV	Tentative ID
1	163, 355	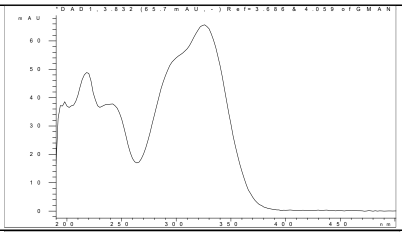	Caffeoyl-quinic acid (chlorogenic acid)
2	291	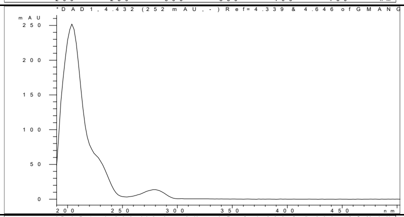	Flavanols (catechin, procyanidin)
3	MS signal too low	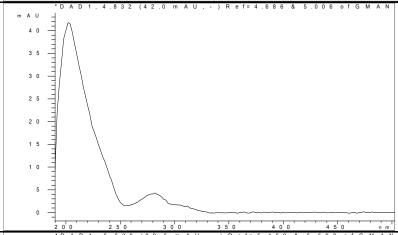	Phenolic (procyanidins)
4	313, 433	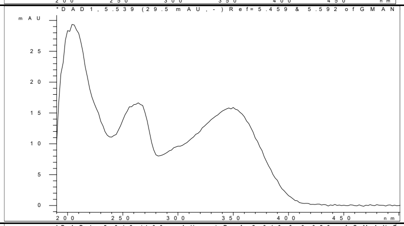	Flavone C-glycoside
5	237	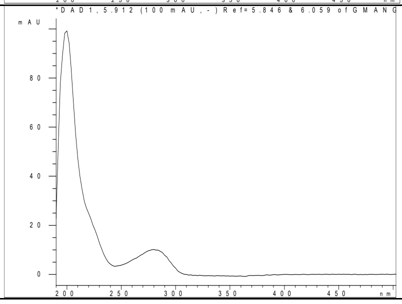	Phenolic

6	327	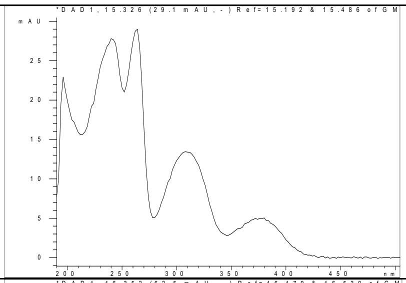	Trihydroxy-prenyl-xanthone
7	381	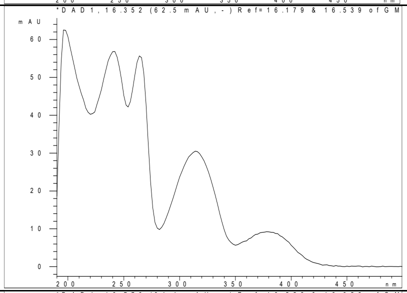	Trihydroxy-triprenyl-xanthone
8	411	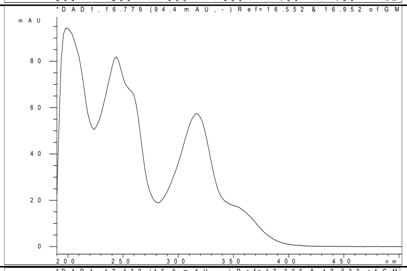	Trihydro-methoxy-diprenyl-xanthone (alpha mangostin)
9	465	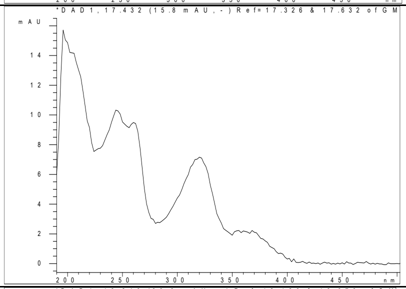	Tetrahydroxy-geranyl-prenyl-xanthone (Garcinone E)
10	409	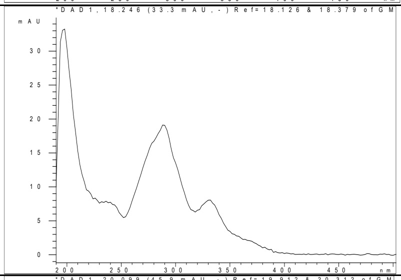	Dihydroxy-prenyl-xanthone-carboxylic acid
11	411	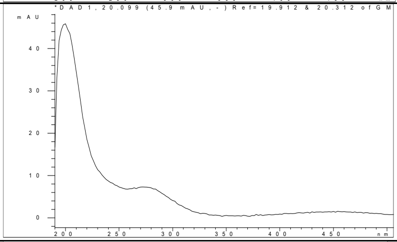	Dihydroxy-dimethyl- trimethyl-tridecatrienyl-benzopyranone (parvifoliol E)
12	397	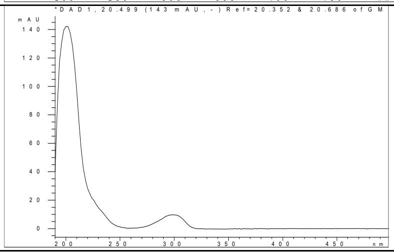	Dihydroxy-dimethyl- trimethyl-tridecatrienyl-benzopyranone (parvifoliol F)

Figure S3. LC-MS chromatogram and UV–visible spectra of *G. mangostana* seeds.

https://doi.org/10.3390/foods15203720

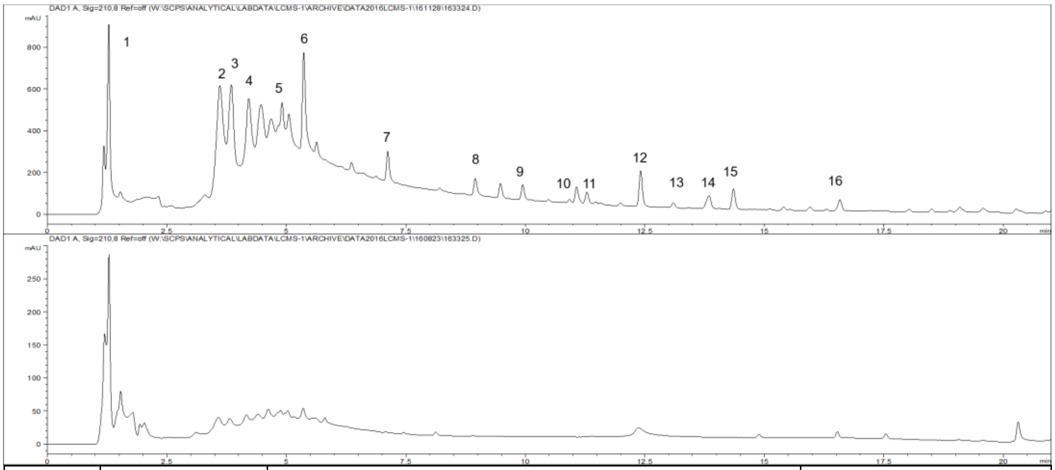

Peak	Fragment ions [M + HI]	UV	Tentative ID
1	193, 209	Supplementary Figure S14	Organic acids (Citric acid, hydroxycitric acid)
2	291, 579	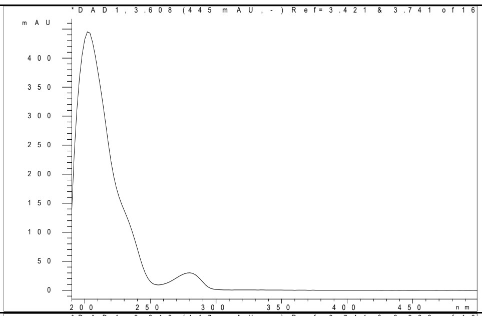	B-type procyanidin dimer (See *G. humilis* procyanidin overlays for more information)
3	291	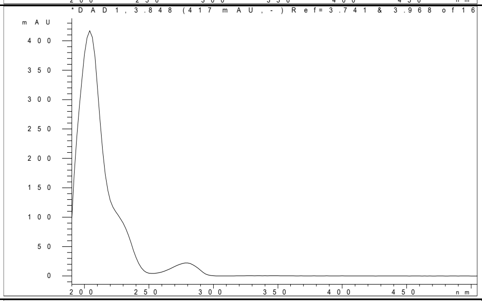	Catechin
4	291, 579	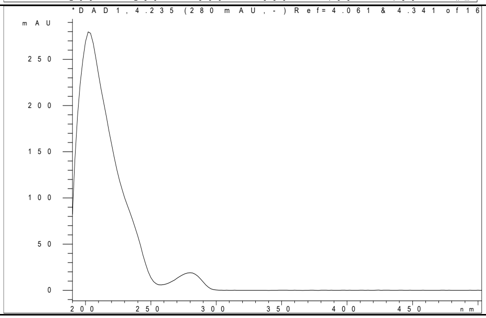	Procyanidin (Supplementary Figure S15)

5	313, 433, 579	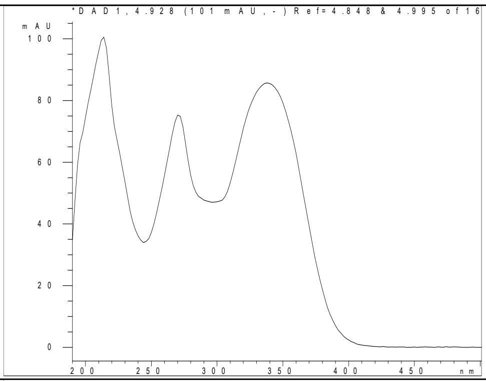	Isovitexin glycoside
6	Supplementary Figure S13	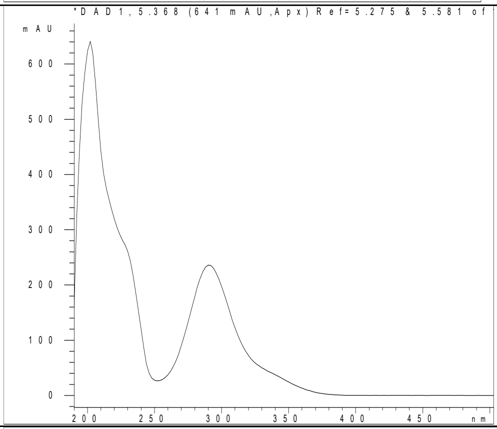	Flavanone derivative
7	543, 705	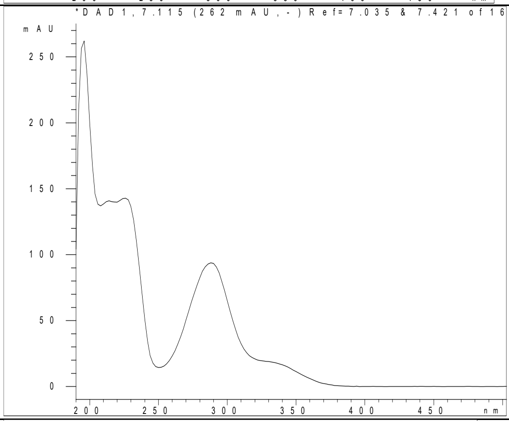	Bi-naringenin glycoside
8	543	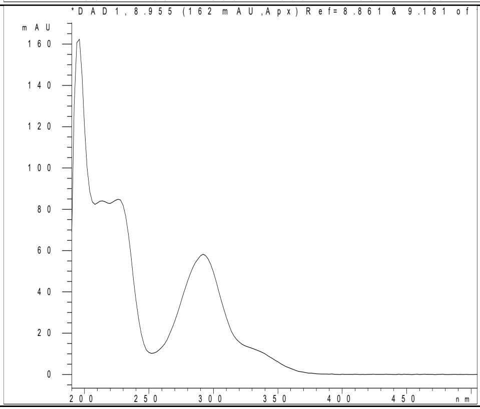	Bi-naringenin

9	273, 329	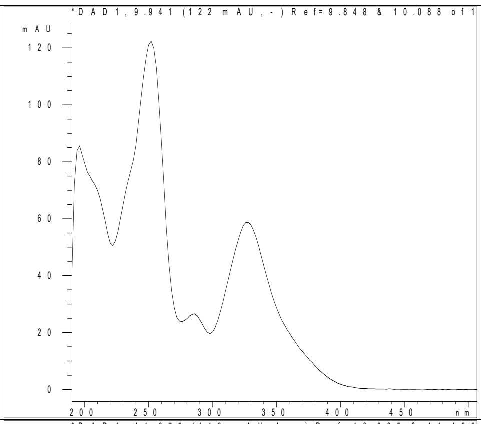	Tetrahydroxy-prenyl-xanthones isomer
10	273, 329	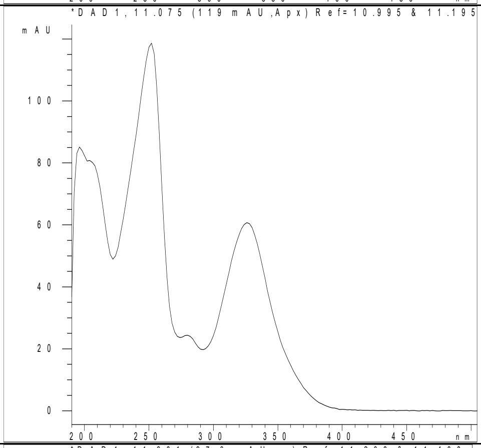	Tetrahydroxy-prenyl-xanthones isomer
11	273, 329	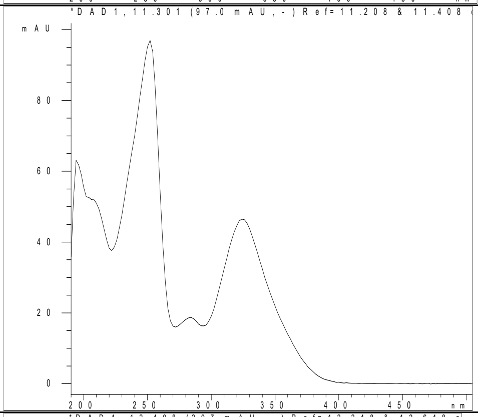	Tetrahydroxy-prenyl-xanthones isomer
12	413, 427	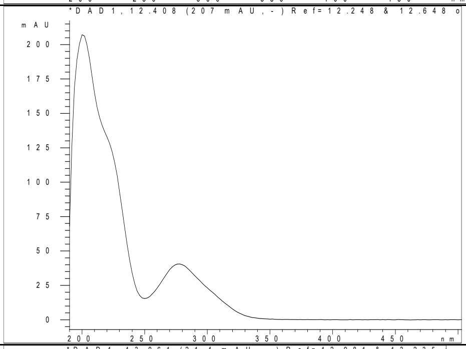	Depsidone derivative
13	-	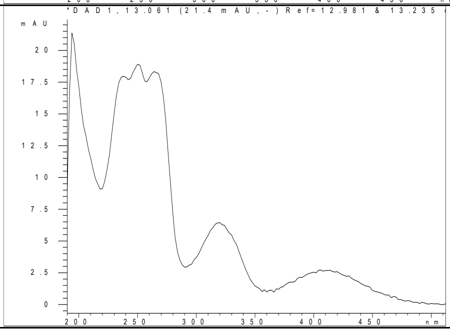	Xanthone derivative

14	273, 285, 341	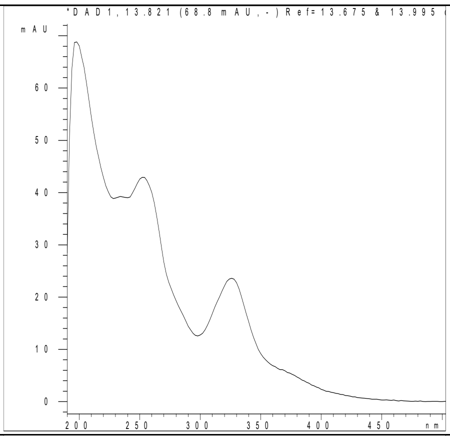	Hydroxy-di-methoxy-prenyl-xanthones
15	273, 397	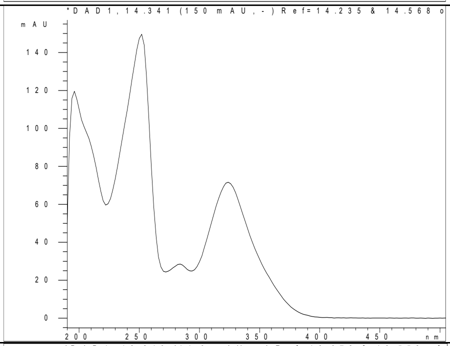	Tetrahydroxy-di-prenyl-xanthone
16	285, 341, 409, 465	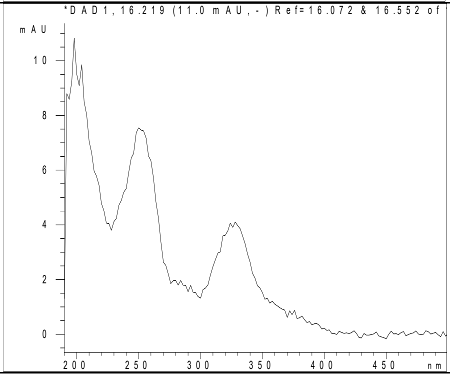	Geranyl-tetrathy-droxy-prenyl-xan-thone isomer

Figure S4. LC-MS chromatogram and UV–visible spectra of *G. humilis* rind.

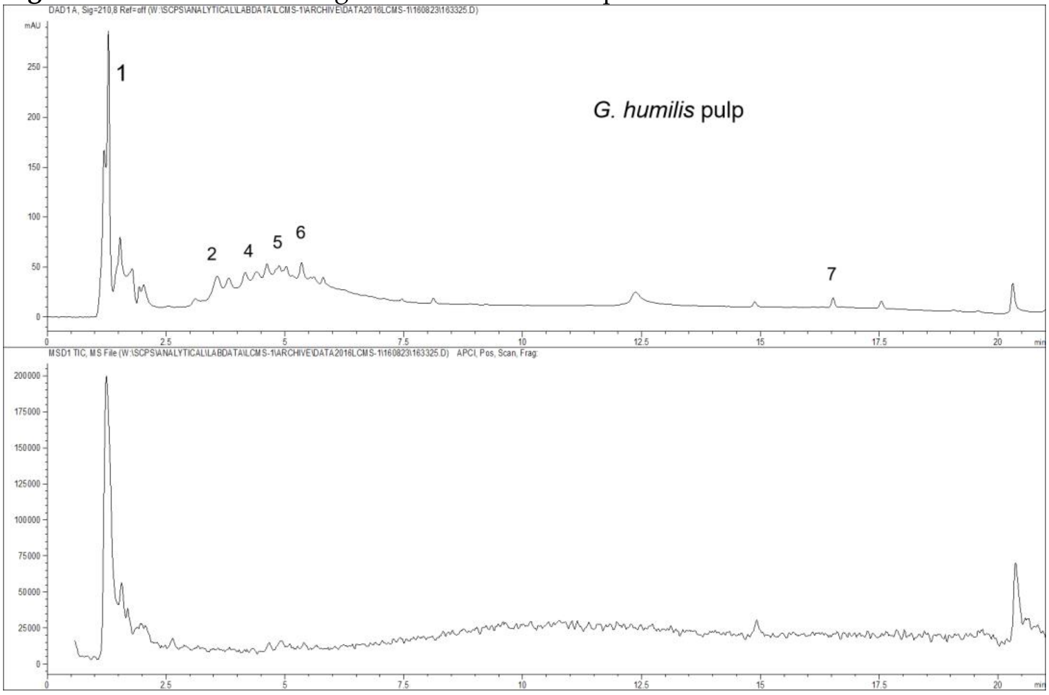

Peak	Fragment ions [M + HI]	UV	Tentative ID
1	193, 209	Supplementary Figure S14	Organic acids (Citric acid, hydroxycitric acid)
2	291, 579	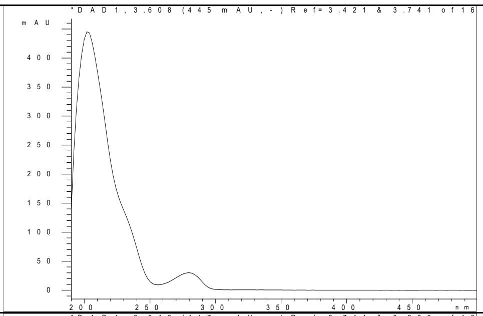	B-type procyanidin dimer (Supplementary Figure S15)
3	291	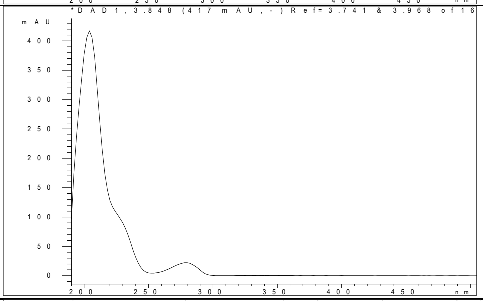	Catechin
4	291, 579	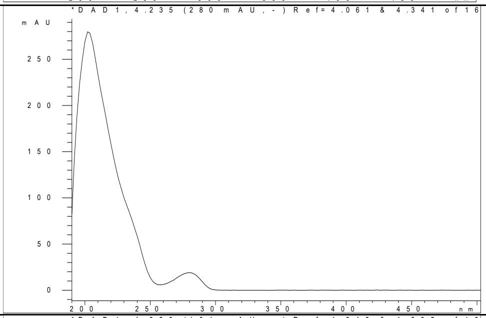	Procyanidin (Supplementary Figure S15)
5	313, 433, 579	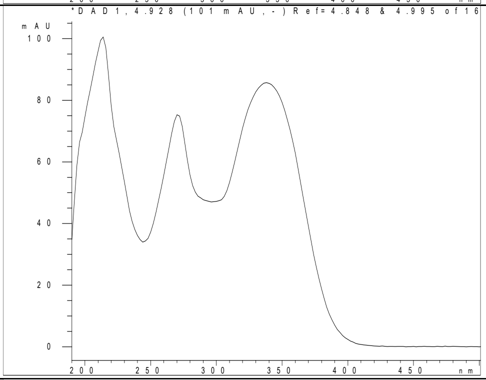	Isovitexin glycoside
6	Supplementary Figure S13	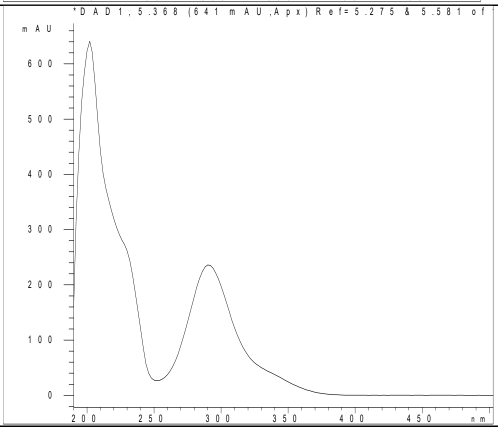	Flavanone derivative

https://doi.org/10.3390/foods15203720

7	285, 341, 409, 465	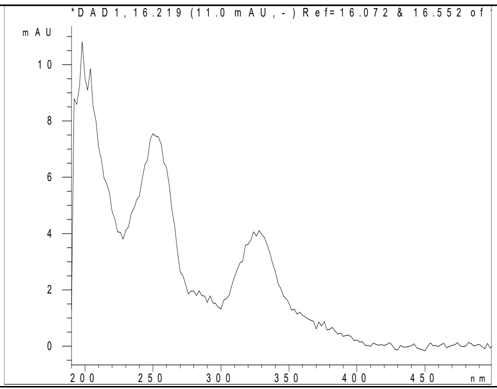	Geranyl-tetrathy-droxy-prenyl-xan-thone isomer

Figure S5. LC-MS chromatogram and UV–visible spectra of *G. humilis* pulp.

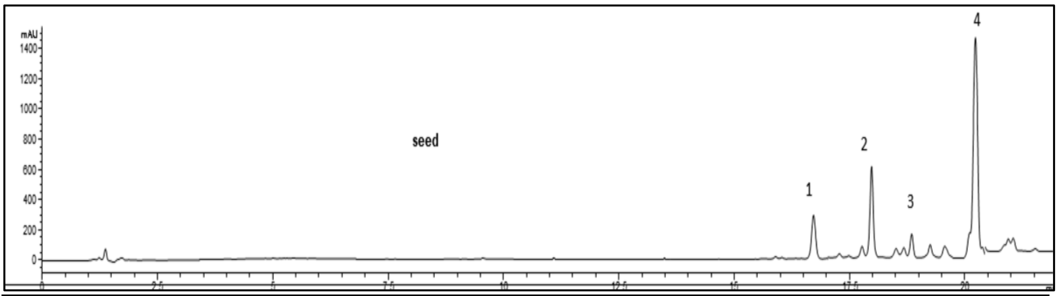

Peak	Fragment ions [M + HI]	UV	Tentative ID
1	343, 399, 411, 467, 479, 535	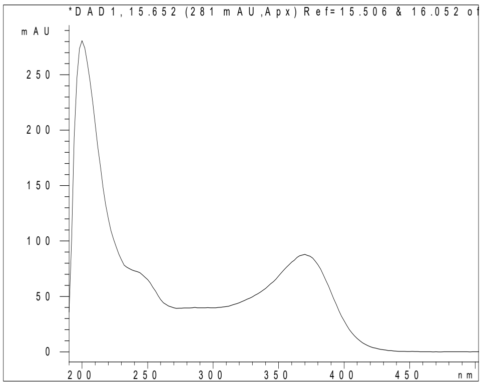	Guttiferone (poly-isoprenylated benzophenone) derivative
2	343, 399, 411, 467, 479, 603	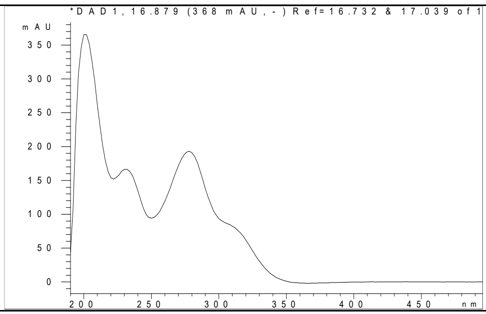	Guttiferone derivative (isogarcinol)
3	327, 395, 451, 519, 587, 601	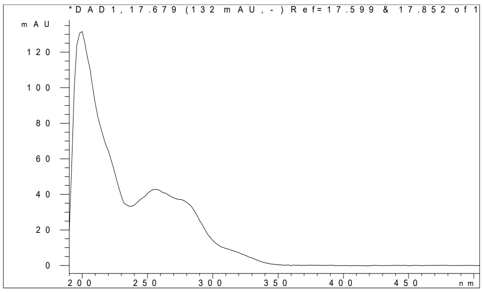	Guttiferone derivative
4	343, 399, 411, 467, 479, 603	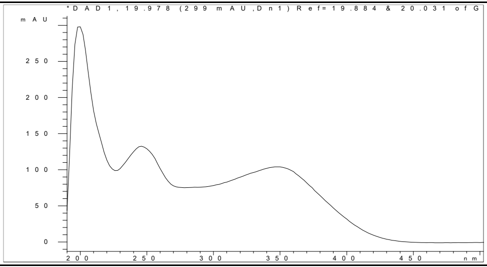	Guttiferone derivative (garcinol)

Figure S6. LC-MS chromatogram and UV–visible spectra of G. humilis seeds.

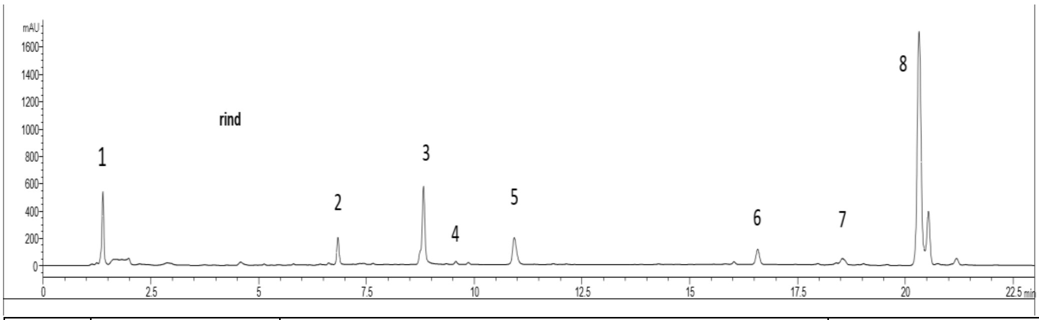

Peak	Fragment ions [M + HI]	UV	Tentative ID
1	193	(see the following *G. dulcis* citric acid overlays)	Citric acid
2	557, 719	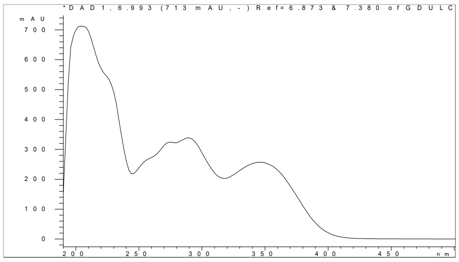	Biflavone glycoside (Morelloflavonne glycoside) (tetrahydroxyflavone-trihydroxyflavone -glucoside)
3	557	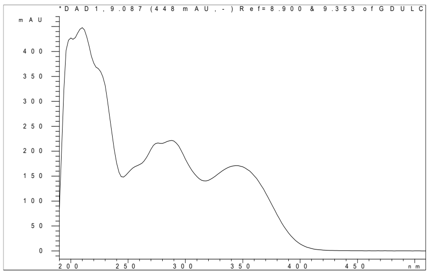	Biflavone (Morelloflavonne) (tetrahydroxyflavone-trihydroxyflavone)
4	541	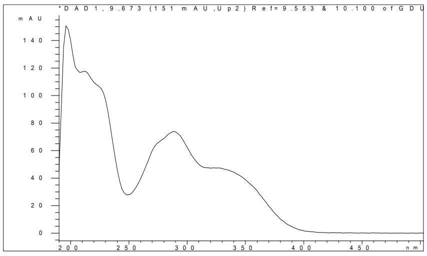	Biflavone (trihydroxyflavone-trihydroxyflavone)
5	557	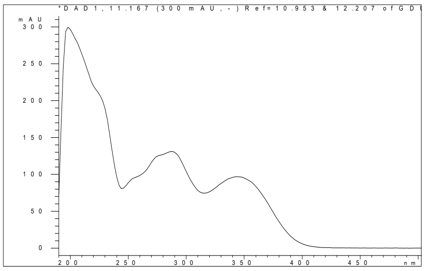	Biflavone (Morelloflavonne) (tetrahydroxyflavone-tetrahydroxyfalvone) derivative
6	Tentative ID based on common UV-Vis spectra	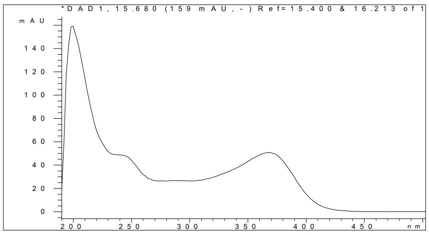	Guttiferone (polyisoprenylated benzophenone) derivative

7	343, 399, 411, 467, 479, 603	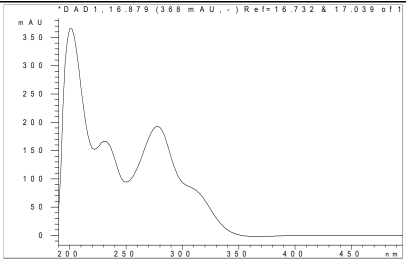	Isogarcinol
8	603	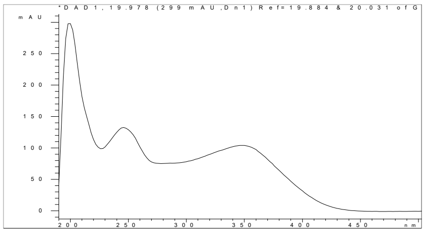	Garcinol

Figure S7. LC-MS chromatogram and UV-visible spectra of *G. dulcis* rind.

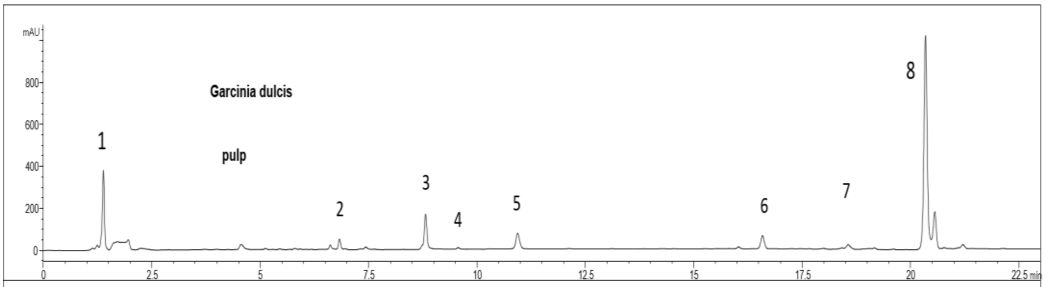

Peak	Fragment ions [M + HI]	UV	Tentative ID
1	193	(see the following *G. dulcis* citric acid overlays)	Citric acid
2	557, 719	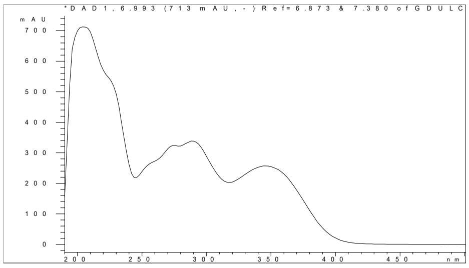	Biflavone glycoside (Morelloflavone glucoside) (tetrahydroxyflavone-trihydroxyflavone - glucoside)
3	557	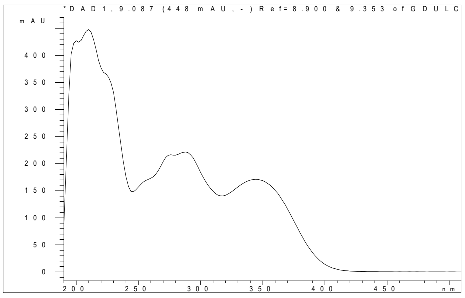	Biflavone (Morelloflavone) (tetrahydroxyflavone-trihydroxyflavone)
4	541	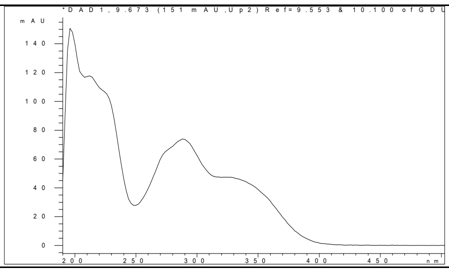	Biflavone (trihydroxyflavone-trihydroxyflavone)

https://doi.org/10.3390/foods15203720

5	557	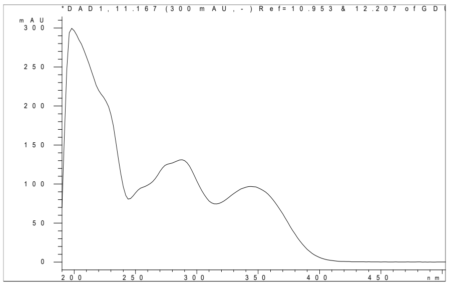	Biflavone (Morelloflavone derivative) (tetrahydroxyflavone-tetrahydroxyfalvone) derivative
6	Tentative ID based on common UV-Vis spectra	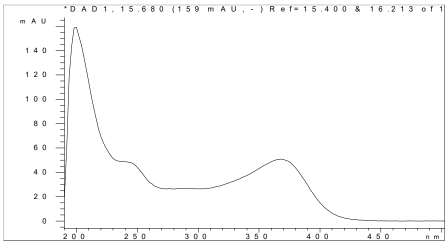	Guttiferone (polyisoprenylated benzophenone) derivative
7	343, 399, 411, 467, 479, 603	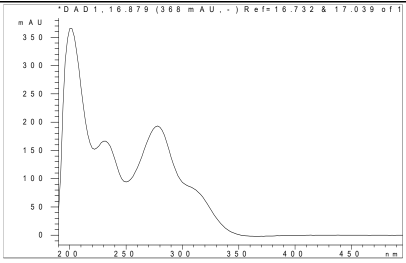	Isogarcinol
8	603	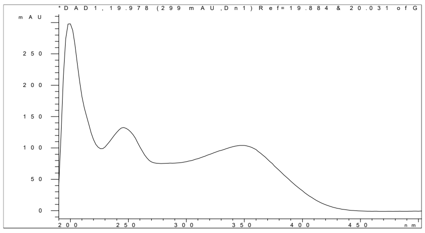	Garcinol

Figure S8. LC-MS chromatogram and UV-visible spectra of *G. dulcis* pulp.

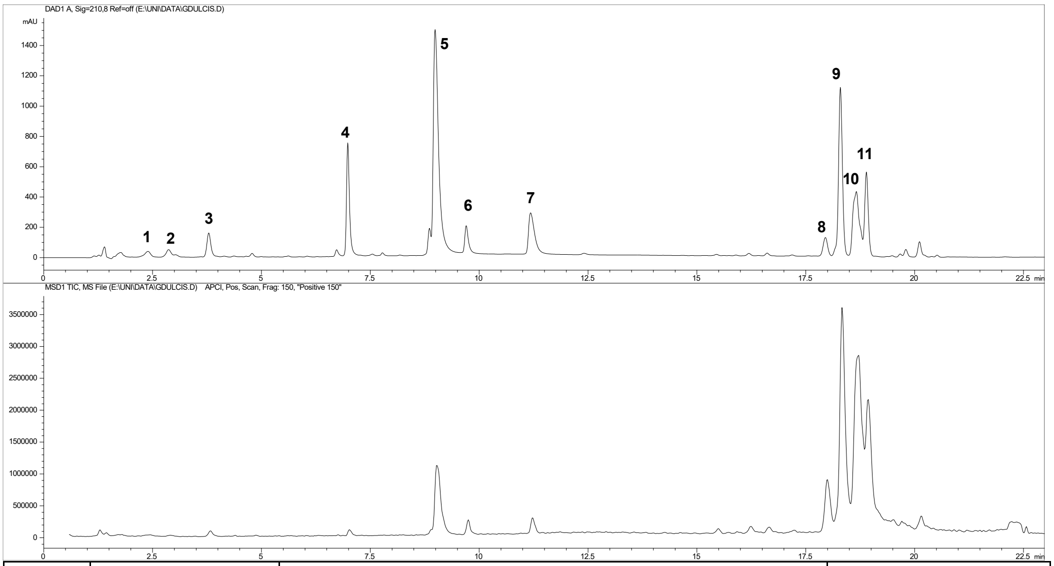

Peak	Fragment ions [M + HI]	UV	Tentative ID
1	144, 162	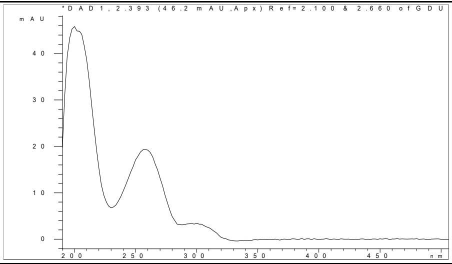	Amine/alkaloid (possibly benzylamine derivative)
2	163, 355	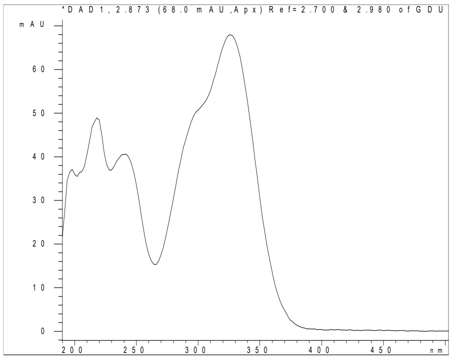	Caffeoyl-quinic acid (chlorogenic acid)
3	119, 147	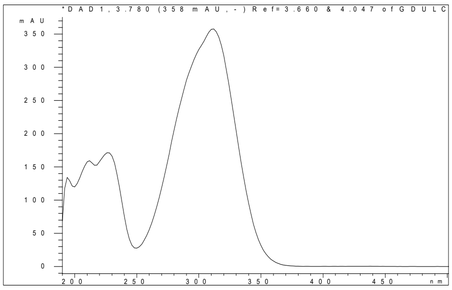	Phenolic acid (coumaric acid)
4	557, 719	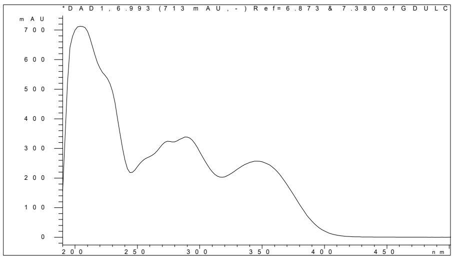	Biflavone glycoside (Morelloflavone glycoside) (tetrahydroxyflavone-trihydroxyflavone - glucoside)

5	557	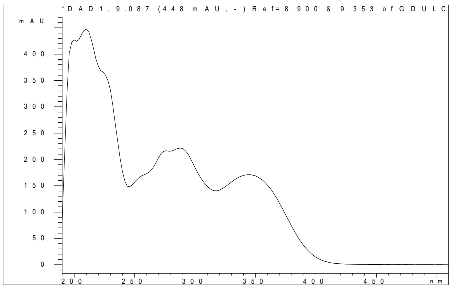	Biflavone (Morelloflavone) (tetrahydroxyflavone-trihydroxyflavone)
6	541	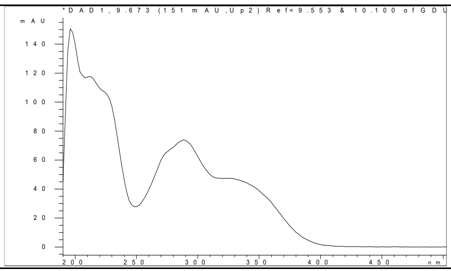	Biflavone (trihydroxyflavone-trihydroxyflavone)
7	557	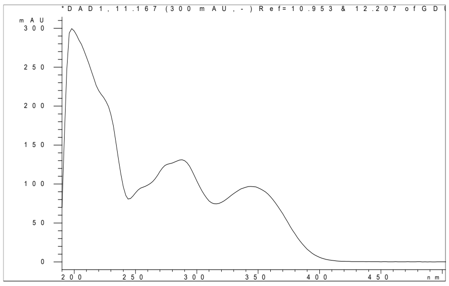	Biflavone ((Morelloflavone derivative) (tetrahydroxyflavone-tetrahydroxyfalvone) derivative
8	485, 517	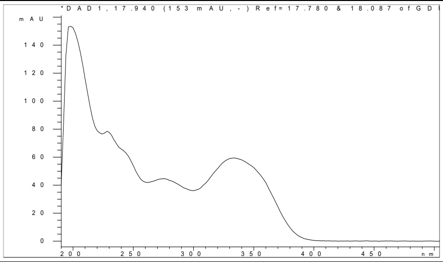	Diprenyl-xanthenone derivative
9	467, 485	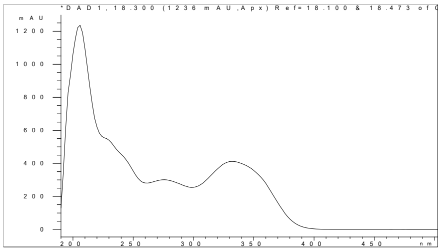	Diprenyl-xanthenone derivative
10	499, 517	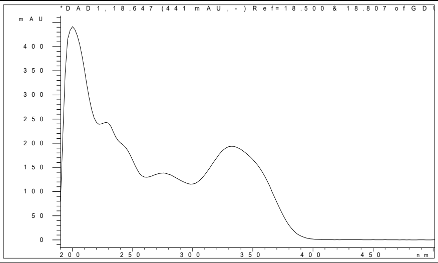	Diprenyl-xanthenone derivative

11	481, 499	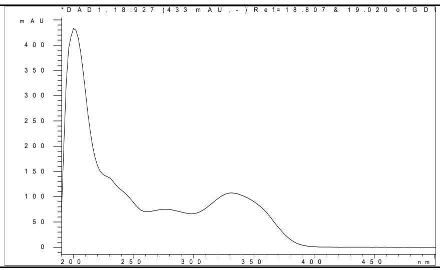	Diprenyl-xanthenone derivative

Figure S9. LC-MS chromatogram and UV-visible spectra of *G. dulcis* seeds.

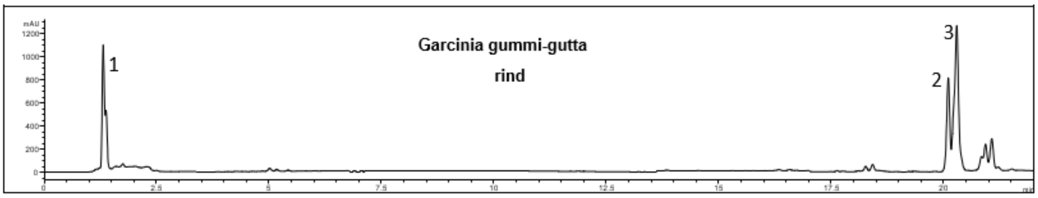

Peak	Fragment ions [M + HI]	UV	Tentative ID
1	193, 209	Supplementary Figure S17	Hydroxycitric/ Citric acid
2	411	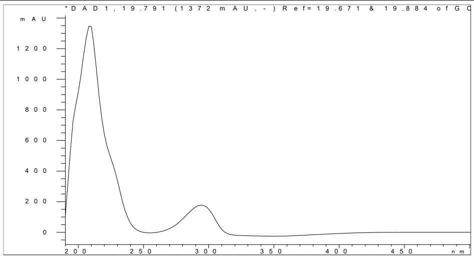	Tocotrienoic acid aldehyde
3	603	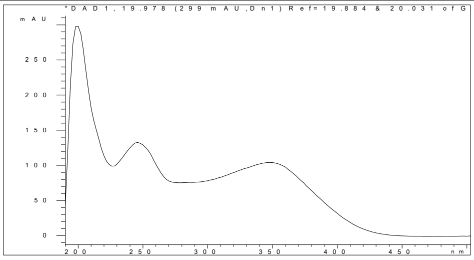	Guttiferone derivative (garcinol)

Figure S10. LC-MS chromatogram and UV-visible spectra of *G. gummi-gutta* rind.

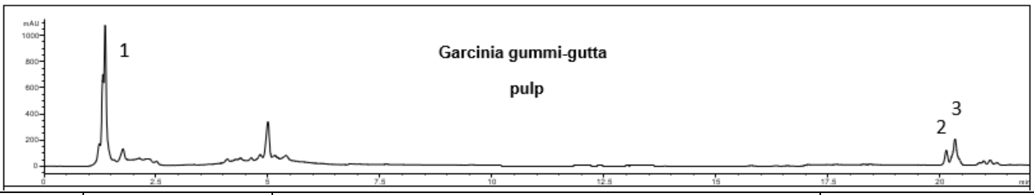

Peak	Fragment ions [M + HI]	UV	Tentative ID
1	193, 209	Supplementary Figure S17	Hydroxycitric / Citric acid
2	411	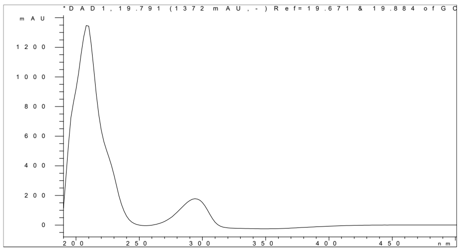	Tocotrienoic acid aldehyde
3	603	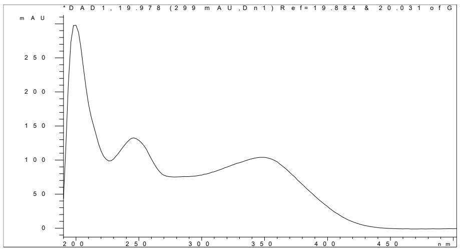	Guttiferone derivative (garcinol)

Figure S11. LC-MS chromatogram and UV-visible spectra of *G. gummi-gutta* seeds.

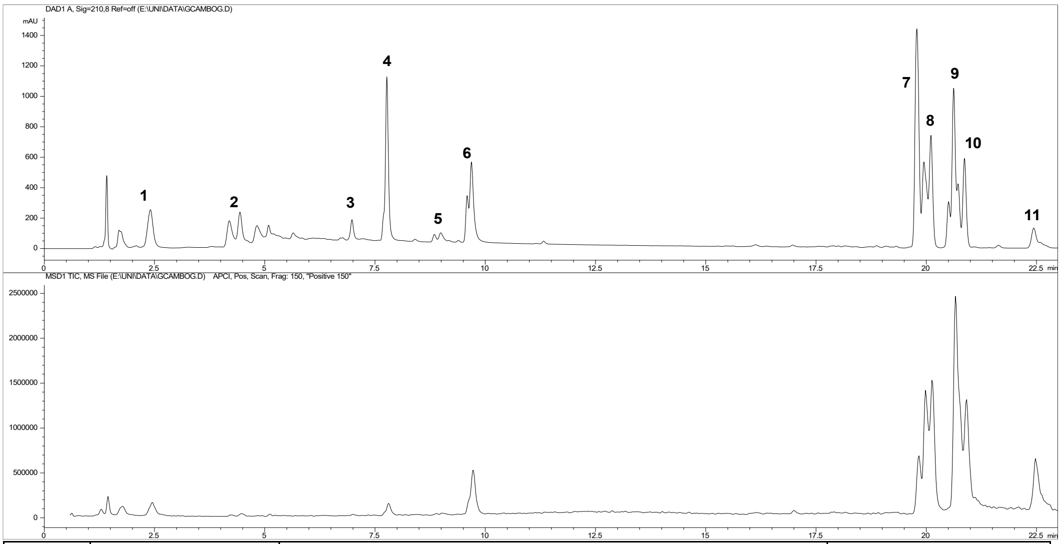

Peak	Fragment ions [M + HI]	UV	Tentative ID
1	144, 162	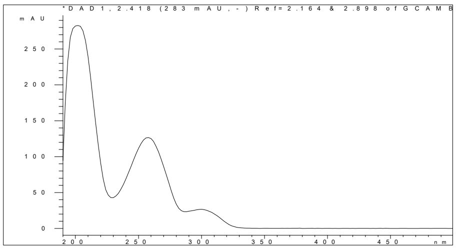	Amine/alkaloid (benzylamine derivative)

2	291, 579	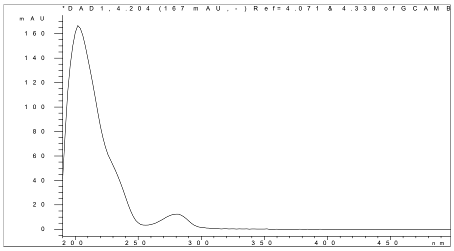	Catechin, procyanidin
3	557, 719	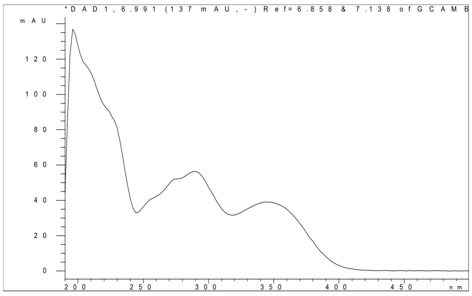	Biflavone glycoside (tetrahydroxyflavone-trihydroxyflavone-glucoside)
4	541, 703	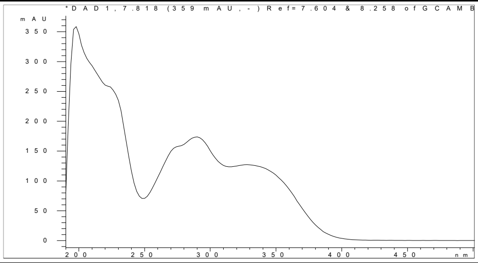	Biflavone glycoside (trihydroxyflavone-trihydroxyflavone-glucoside)
5	557	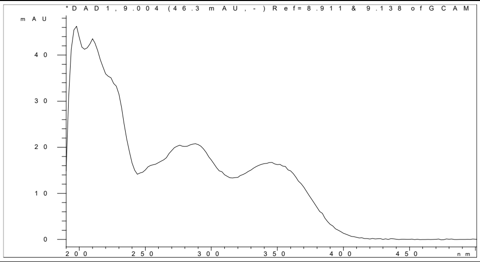	Biflavone (tetrahydroxyflavone-trihydroxyflavone)
6	541	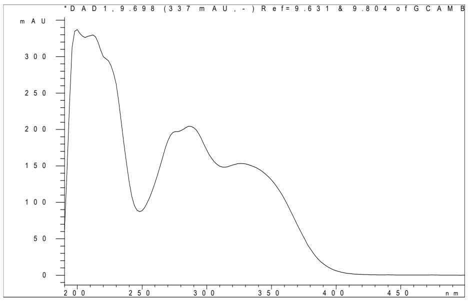	Biflavone (trihydroxyflavone-trihydroxyflavone)
7	411	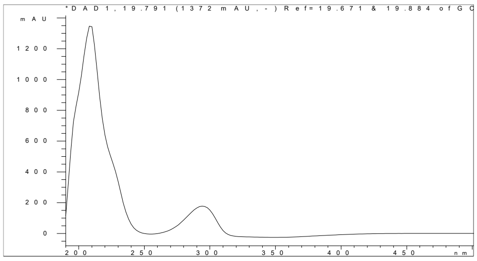	Tocotrienoic acid aldehyde

https://doi.org/10.3390/foods15203720

8	603	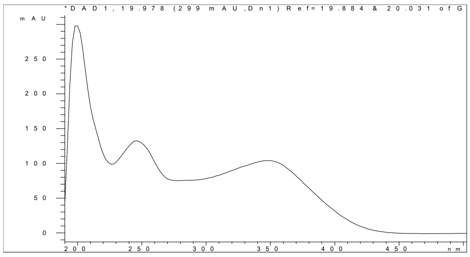	Guttiferone derivative (garcinol)
9	587	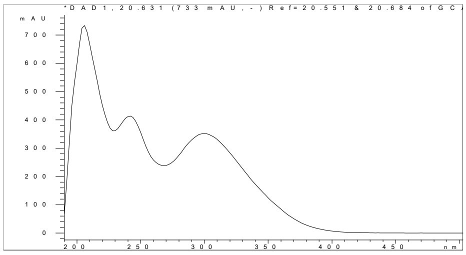	Deoxy-guttiferone derivative
10	587	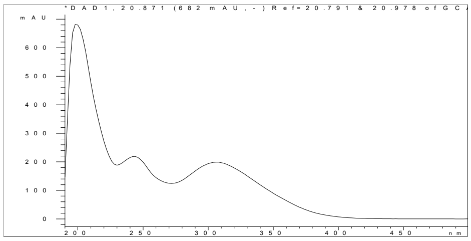	Deoxy-guttiferone derivative
11	571	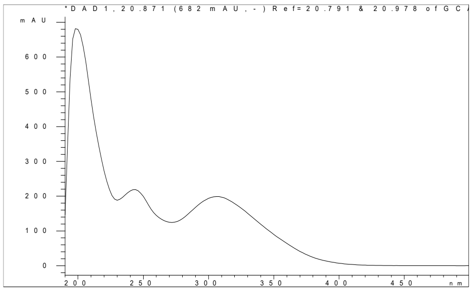	Di-deoxy-guttiferone derivative

Figure S12. LC-MS chromatogram and UV-visible spectra of *G. gummi-gutta* seeds.

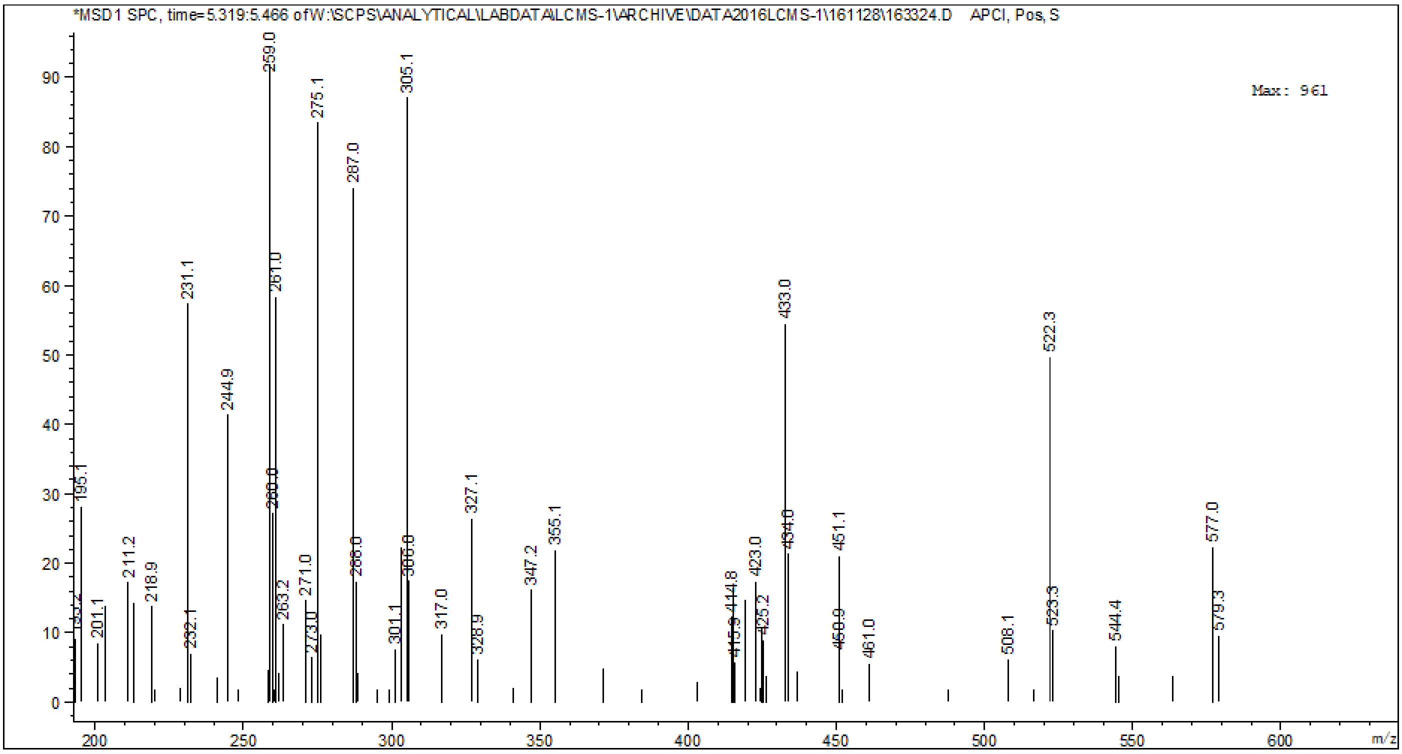

Figure S13. *Garcinia humilis* peak #6.

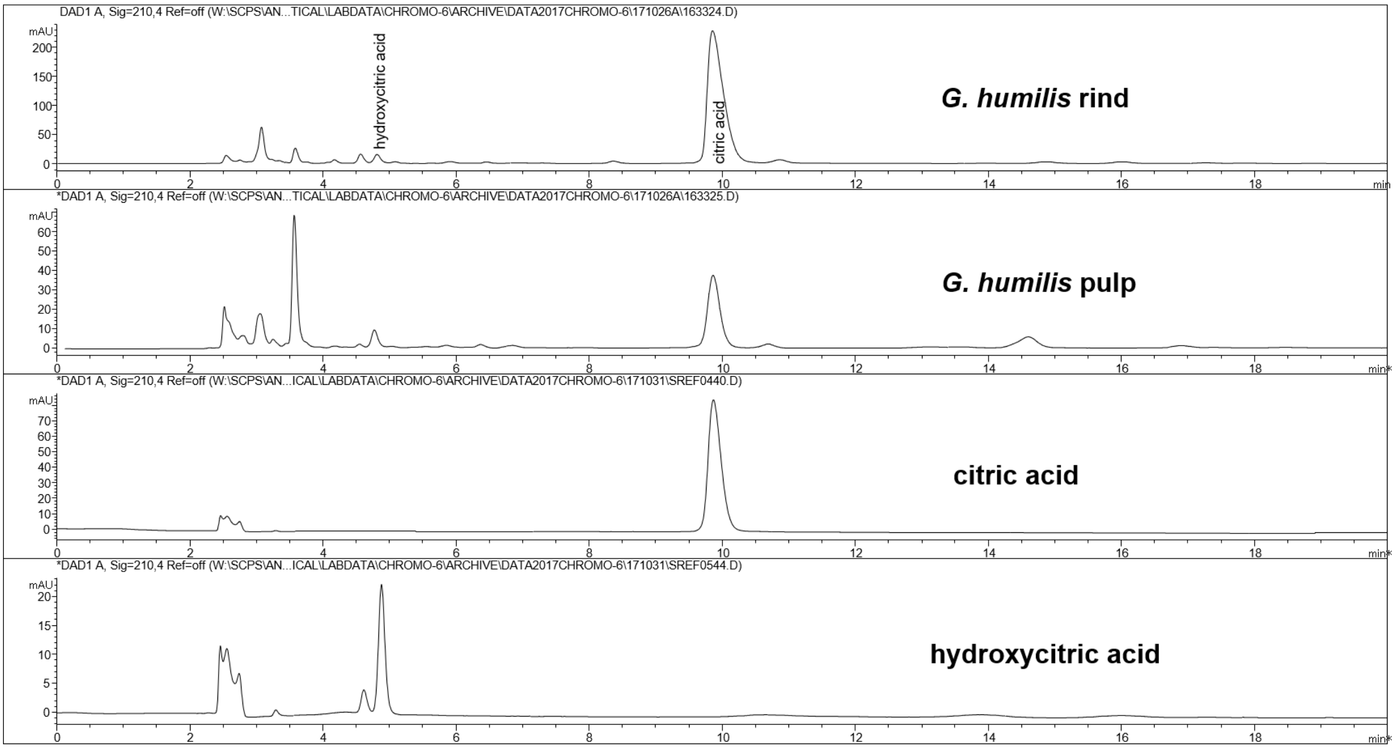

Figure S14. *G. humilis* organic acid chromatogram.

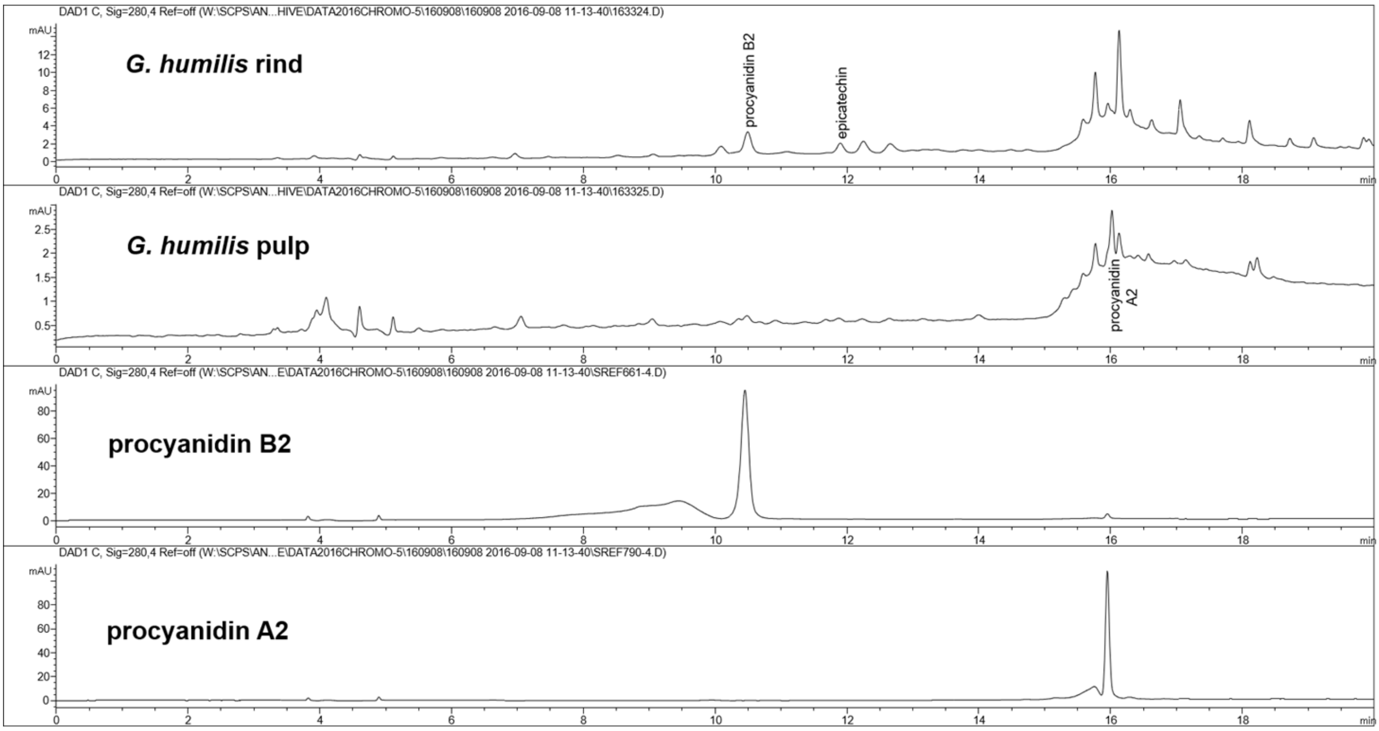

Figure S15. *G. humilis* procyanidin chromatogram.

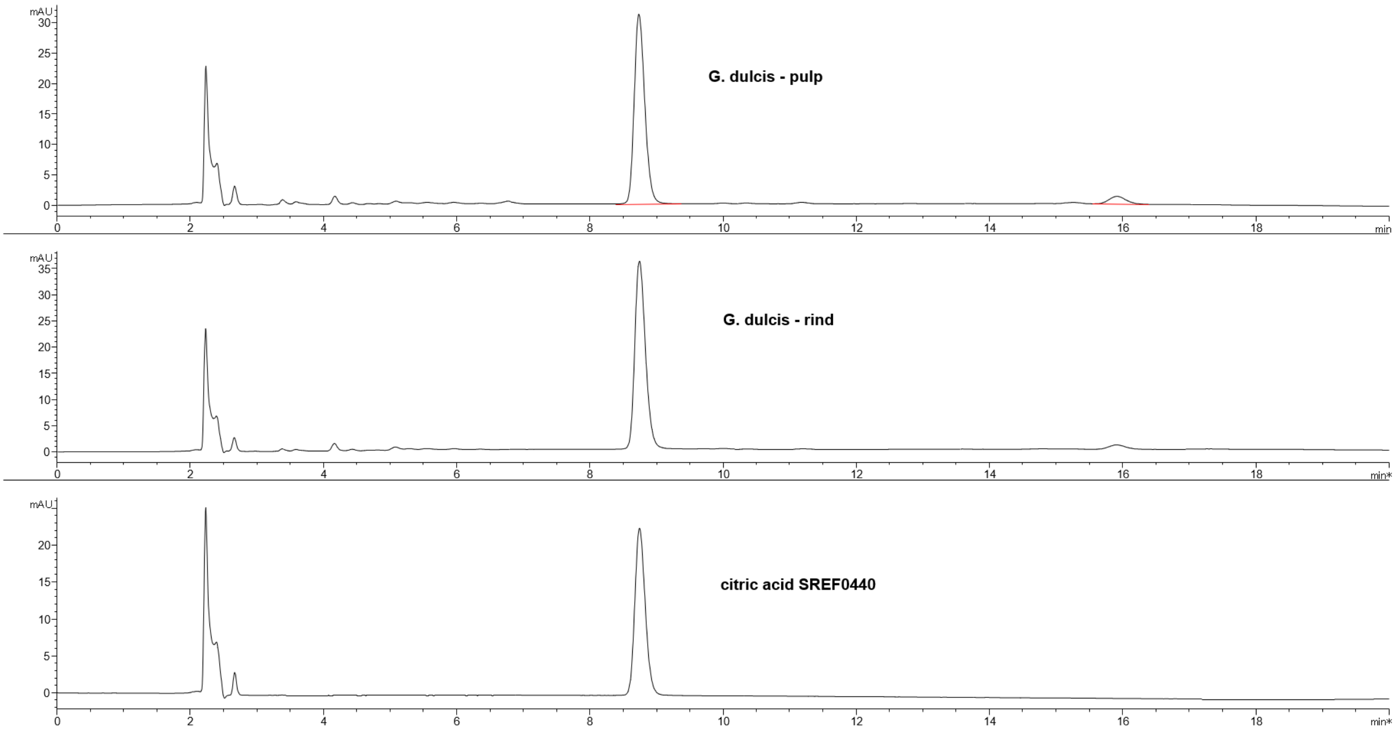

Figure S16. *G. dulcis* citric acid overlays chromatogram.

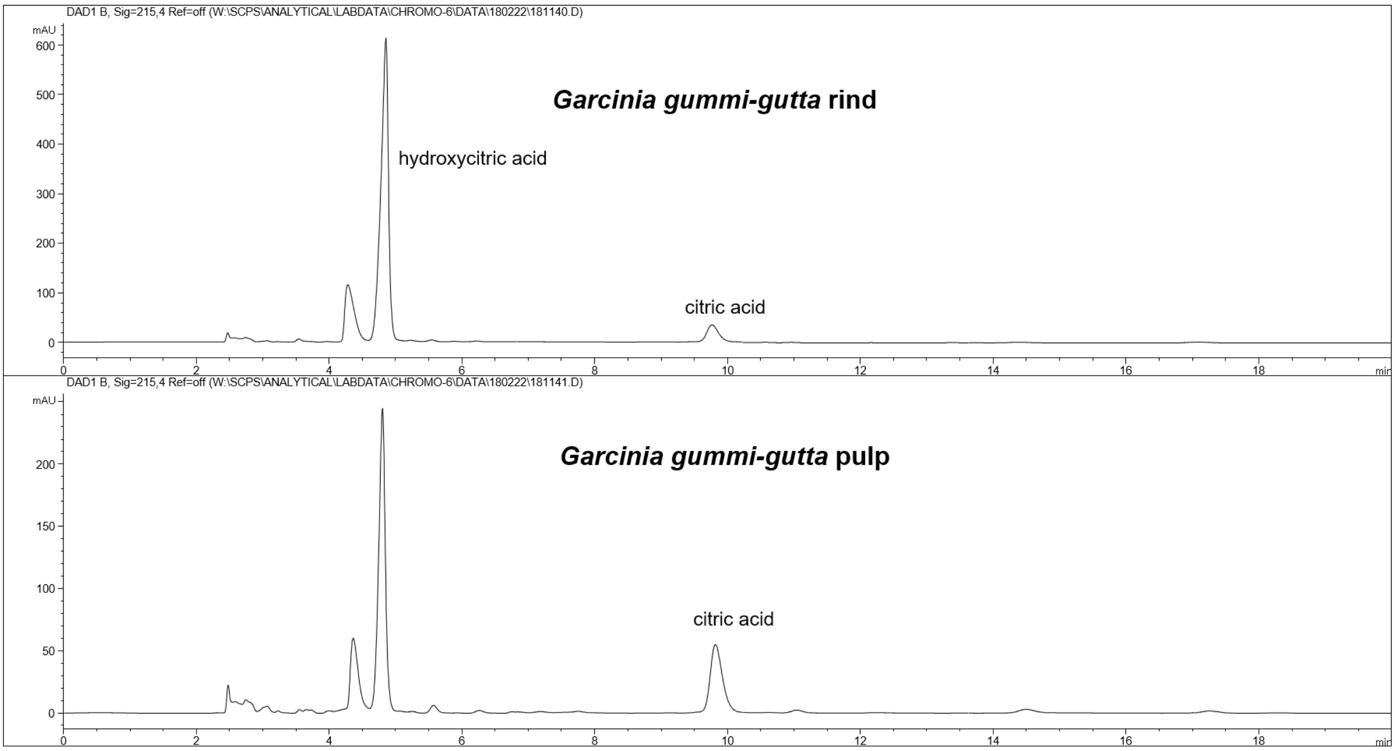

Figure S17. *G. gummi-gutta* chromatogram – organic acid overlays.

Method C:\DATA\CHROMO 6\ANQAPROCYA9.M

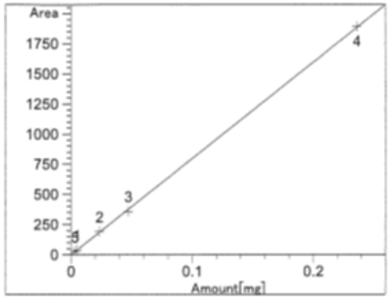

procyanidin B2 at exp. RT: 10.460
DAD1 C, Sig=280,4 Ref=off
Correlation: 0.99994
Residual Std. Dev.: 10.89165
Formula: y = mx
m: 7994.27699
x: Amount
y: Area

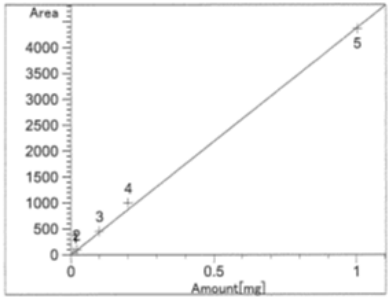

epicatechin at exp. RT: 11.906
DAD1 C, Sig=280,4 Ref=off
Correlation: 0.99963
Residual Std. Dev.: 60.82830
Formula: y = mx
m: 4380.34207
x: Amount
y: Area

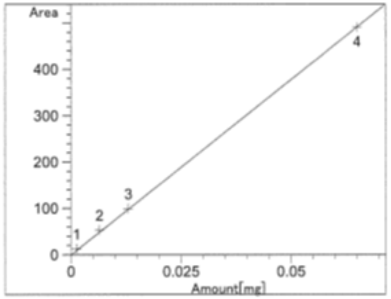

procyanidin A2 at exp. RT: 15.950
DAD1 C, Sig=280,4 Ref=off
Correlation: 0.99996
Residual Std. Dev.: 2.68594
Formula: y = mx
m: 7562.26469
x: Amount
y: Area

Calibration Table

Calib. Data Modified : 14 September, 2016 11:55:48 AM

Rel. Reference Window : 3.000 %
Abs. Reference Window : 0.000 min
Rel. Non-ref. Window : 3.000 %
Abs. Non-ref. Window : 0.000 min
Uncalibrated Peaks : using compound procyanidin B2
Partial Calibration : Yes, identified peaks are recalibrated
Correct All Ret. Times: No, only for identified peaks

Curve Type : Linear
Origin : Forced
Weight : Equal

Recalibration Settings:
Average Response : Average all calibrations
Average Retention Time: Floating Average New 75%

Calibration Report Options :
Printout of recalibrations within a sequence:
Calibration Table after Recalibration
Normal Report after Recalibration
If the sequence is done with bracketing:
Results of first cycle (ending previous bracket)

Signal 1: DAD1 C, Sig=280,4 Ref=off

RetTime [min]	Sig	Lvl	Amount [mg]	Area	Amt/Area	Ref	Grp	Name
7.754	1	1	1.66149e-3	8.81715	1.88439e-4		1	deriv 1
		2	7.07411e-3	34.72487	2.03719e-4			
		3	1.78011e-2	89.73521	1.98374e-4			
		4	3.60464e-2	179.81177	2.00468e-4			
8.733	1	1	4.38506e-4	2.32704	1.88439e-4		1	deriv 2
		2	1.32589e-3	6.50842	2.03719e-4			
		3	3.19890e-3	16.12561	1.98374e-4			
		4	5.95356e-3	29.69835	2.00468e-4			
9.603	1	1	1.00200e-2	43.10415	2.32460e-4			catechin
		2	2.00400e-2	88.37025	2.26773e-4			
		3	1.00200e-1	449.67938	2.22825e-4			
		4	2.00400e-1	996.42664	2.01119e-4			
		5	1.00200	4364.32813	2.29589e-4			
10.460	1	5	3.20000e-3	21.78440	1.46894e-4			procyanidin B2
		1	4.72000e-3	36.01475	1.31057e-4			
		2	2.36000e-2	188.96396	1.24892e-4			
		3	4.72000e-2	356.37711	1.32444e-4			
		4	2.36000e-1	1890.89587	1.24809e-4			
11.906	1	1	1.00200e-2	43.10400	2.32461e-4			epicatechin
		2	2.00400e-2	88.37000	2.26774e-4			
		3	1.00200e-1	449.68000	2.22825e-4			
		4	2.00400e-1	996.43000	2.01118e-4			
		5	1.00200	4364.30000	2.29590e-4			
15.950	1	1	1.30000e-3	12.39630	1.04870e-4			procyanidin A2
		2	6.50000e-3	52.85712	1.22973e-4			
		3	1.30000e-2	99.29494	1.30923e-4			
		4	6.50000e-2	490.92856	1.32402e-4			

Figure S18. Calibration curves: procyanidins.

Method W:\SCPS\ANALYTICAL\LABDATA\CHROMO-6\METHODS\QAORGANICACID1.M

Calibration Curves

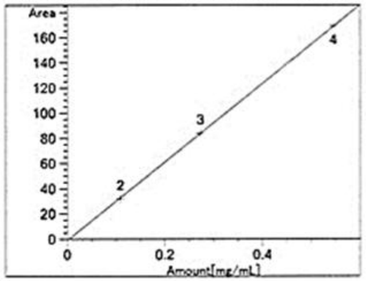

Quinic acid at exp. RT: 3.690
DAD1 A, Sig=210,4 Ref=off
Correlation: 0.99992
Residual Std. Dev.: 1.11143
Formula: y = mx + b
m: 311.45005
b: -9.33721e-1
x: Amount
y: Area

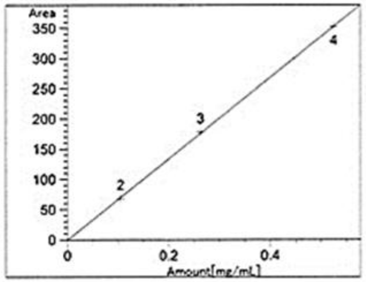

Citric acid at exp. RT: 9.858
DAD1 A, Sig=210,4 Ref=off
Correlation: 0.99994
Residual Std. Dev.: 2.07158
Formula: y = mx + b
m: 673.89905
b: -7.03543e-1
x: Amount
y: Area

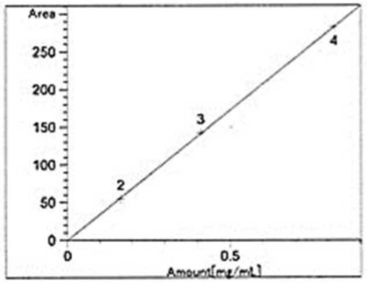

Mallic acid at exp. RT: 11.181
DAD1 A, Sig=210,4 Ref=off
Correlation: 0.99996
Residual Std. Dev.: 1.31734
Formula: y = mx + b
m: 345.93853
b: -4.38316e-1
x: Amount
y: Area

Method W:\SCPS\ANALYTICAL\LABDATA\CHROMO-6\METHODS\QAORGANICACID1.M

Calibration Table

Calib. Data Modified : 9/11/2017 3:29:06 PM

Rel. Reference Window : 5.000 %
Abs. Reference Window : 0.000 min
Rel. Non-ref. Window : 5.000 %
Abs. Non-ref. Window : 0.000 min
Uncalibrated Peaks : not reported
Partial Calibration : Yes, identified peaks are recalibrated
Correct All Ret. Times: No, only for identified peaks

Curve Type : Linear
Origin : Included
Weight : Equal

Recalibration Settings:
Average Response : Average all calibrations
Average Retention Time: Floating Average New 75%

Calibration Report Options :
Printout of recalibrations within a sequence:
Calibration Table after Recalibration
Normal Report after Recalibration
If the sequence is done with bracketing:
Results of first cycle (ending previous bracket)

Signal 1: DAD1 A, Sig=210,4 Ref=off

RetTime [min]	Sig	Lvl	Amount [mg/mL]	Area	Amt/Area	Ref	Grp	Name
3.690	1	2	1.09000e-1	31.76996	3.43091e-3			Quinic acid
		3	2.72500e-1	84.05997	3.24173e-3			
		4	5.45000e-1	168.99365	3.22497e-3			
9.858	1	2	1.05000e-1	67.99652	1.54420e-3			Citric acid
		3	2.62500e-1	178.08281	1.47403e-3			
		4	5.25000e-1	352.56140	1.48910e-3			
11.181	1	2	1.64000e-1	54.99214	2.98224e-3			Mallic acid
		3	4.10000e-1	142.60539	2.87507e-3			
		4	8.20000e-1	282.88751	2.89868e-3			

Group summary :

Group 1 (crocins) :
Group members:
Group Amount Calculation:
Level 1 with amount 0.33600 mg/mL
Level 2 with amount 0.84000 mg/mL
Level 3 with amount 1.68000 mg/mL
Level 4 with amount 3.32000 mg/mL

Peak Sum Table

No Entries in table

Figure S19. Calibration curves: organic acids (quinic acid, citric acid, malic acid).

Calibration Curves

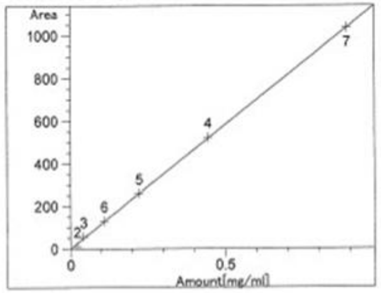

HCA lactone at exp. RT: 4.200
DAD1 B, Sig=215,4 Ref=off
Correlation: 0.99991
Residual Std. Dev.: 7.14579
Formula: y = mx
m: 1163.00280
x: Amount
y: Area

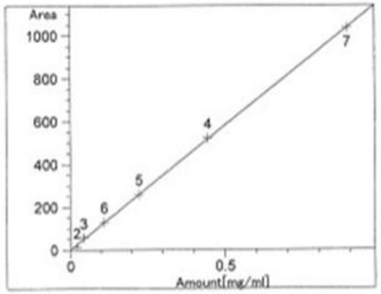

HCA at exp. RT: 4.900
DAD1 B, Sig=215,4 Ref=off
Correlation: 0.99991
Residual Std. Dev.: 7.14666
Formula: y = mx
m: 1162.98670
x: Amount
y: Area

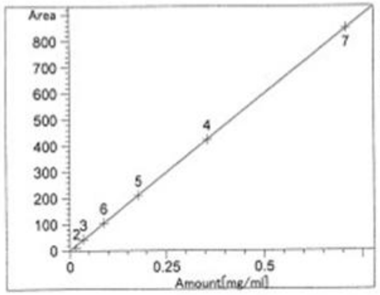

citric acid at exp. RT: 9.800
DAD1 B, Sig=215,4 Ref=off
Correlation: 0.99992
Residual Std. Dev.: 5.65992
Formula: y = mx
m: 1197.44284
x: Amount
y: Area

Calibration Table

Calib. Data Modified : 26/02/2018 3:24:47 PM

Rel. Reference Window : 5.000 %
Abs. Reference Window : 0.000 min
Rel. Non-ref. Window : 5.000 %
Abs. Non-ref. Window : 0.000 min
Uncalibrated Peaks : not reported
Partial Calibration : Yes, identified peaks are recalibrated
Correct All Ret. Times: No, only for identified peaks

Curve Type : Linear
Origin : Forced
Weight : Equal

Recalibration Settings:
Average Response : Average all calibrations
Average Retention Time: Floating Average New 75%

Calibration Report Options :
Printout of recalibrations within a sequence:
Calibration Table after Recalibration
Normal Report after Recalibration
If the sequence is done with bracketing:
Results of first cycle (ending previous bracket)

Signal 1: DAD1 B, Sig=215,4 Ref=off

RetTime [min]	Sig	Lvl	Amount [mg/ml]	Area	Amt/Area	Ref	Grp	Name
4.200	1	2	2.22500e-2	10.89400	2.04241e-3			HCA lactone
		3	4.45000e-2	56.20800	7.91702e-4			
		6	1.11250e-1	129.60000	8.58410e-4			
		5	2.22500e-1	259.35000	8.57914e-4			
		4	4.45000e-1	520.44000	8.55046e-4			
		7	8.90000e-1	1033.60000	8.61068e-4			
4.900	1	2	2.22500e-2	10.89402	2.04240e-3			HCA
		3	4.45000e-2	56.20790	7.91704e-4			
		6	1.11250e-1	129.60350	8.58387e-4			
		5	2.22500e-1	259.35263	8.57905e-4			
		4	4.45000e-1	520.44000	8.55046e-4			
		7	8.90000e-1	1033.57983	8.61085e-4			
9.800	1	2	1.77500e-2	8.70775	2.03841e-3			citric acid
		3	3.55000e-2	43.13294	8.23037e-4			
		6	8.87500e-2	105.73306	8.39378e-4			
		5	1.77500e-1	211.48676	8.39296e-4			
		4	3.55000e-1	424.62000	8.36042e-4			
		7	7.10000e-1	851.03534	8.34278e-4			

Peak Sum Table

No Entries in table

Figure S20. Calibration curves: hydroxycitric acid.

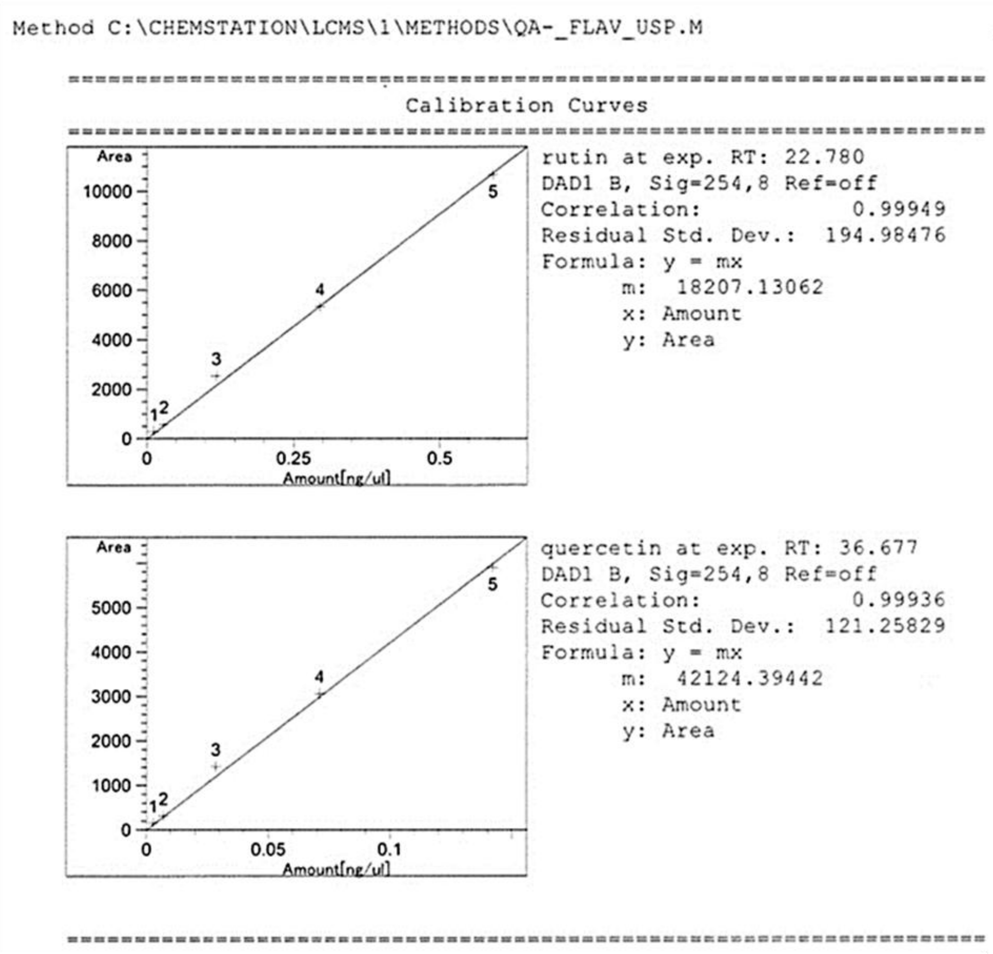

Method C:\CHEMSTATION\LCMS\1\METHODS\QA-_FLAV_USP.M

Calibration Table

Calib. Data Modified : 8/05/2017 9:38:19 AM

Rel. Reference Window : 5.000 %
Abs. Reference Window : 0.000 min
Rel. Non-ref. Window : 5.000 %
Abs. Non-ref. Window : 0.000 min
Uncalibrated Peaks : Separately calculated (see below)
Partial Calibration : Yes, identified peaks are recalibrated
Correct All Ret. Times: No, only for identified peaks

Curve Type : Linear
Origin : Forced
Weight : Equal

Recalibration Settings:
Average Response : Average all calibrations
Average Retention Time: Floating Average New 75%

Calibration Report Options :
Printout of recalibrations within a sequence:
Calibration Table after Recalibration
Normal Report after Recalibration
If the sequence is done with bracketing:
Results of first cycle (ending previous bracket)

Signal 1: DAD1 A, Sig=210,8 Ref=off
Uncalibrated Peaks : using compound quercetin
Signal 2: MSD1 TIC, MS File, Pos, Scan, Frag: 150, "Positive 150"
Uncalibrated Peaks : not reported
Signal 3: DAD1 B, Sig=254,8 Ref=off
Uncalibrated Peaks : using compound quercetin

RetTime [min]	Sig	Lvl	Amount [ng/ul]	Area	Amt/Area	Ref	Grp	Name
22.780	3	1	1.18000e-2	268.83411	4.38932e-5			rutin
		2	2.95000e-2	569.77985	5.17744e-5			
		3	1.18000e-1	2526.77710	4.66998e-5			
		4	2.95000e-1	5334.00098	5.53056e-5			
		5	5.90000e-1	1.06824e4	5.52311e-5			
36.677	3	1	2.80000e-3	143.19781	1.95534e-5			quercetin
		2	7.10000e-3	307.68253	2.30757e-5			
		3	2.84000e-2	1413.23987	2.00957e-5			
		4	7.10000e-2	3060.21045	2.32010e-5			
		5	1.42000e-1	5902.66553	2.40569e-5			

Peak Sum Table

No Entries in table

Figure S21. Calibration curves: flavonoids (rutin and quercetin).

Method D:\LCMS-2\METHODS\ANGARCINOLXANTHONE.M

Calibration Curves

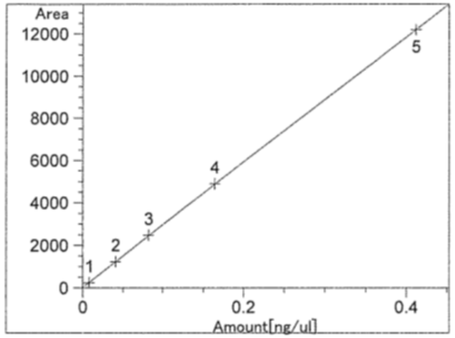

a-mangostin at exp. RT: 7.446
DAD1 B, Sig=240,4 Ref=off
Correlation: 0.99999
Residual Std. Dev.: 18.79491
Formula: y = mx + b
m: 29569.03444
b: 10.29081
x: Amount
y: Area

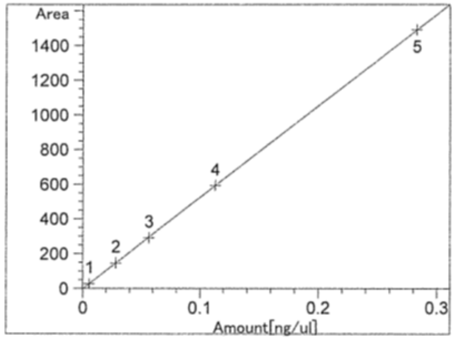

garcinol at exp. RT: 15.157
DAD1 B, Sig=240,4 Ref=off
Correlation: 0.99999
Residual Std. Dev.: 2.98131
Formula: y = mx + b
m: 5279.03913
b: -3.86958
x: Amount
y: Area

Calibration Table

Calib. Data Modified : Tuesday, 17 October 2017 8:42:57 AM

Rel. Reference Window : 5.000 %
Abs. Reference Window : 0.000 min
Rel. Non-ref. Window : 5.000 %
Abs. Non-ref. Window : 0.000 min
Uncalibrated Peaks : not reported
Partial Calibration : Yes, identified peaks are recalibrated
Correct All Ret. Times: No, only for identified peaks

Curve Type : Linear
Origin : Included
Weight : Equal

Recalibration Settings:
Average Response : Average all calibrations
Average Retention Time: Floating Average New 75%

Calibration Report Options :
Printout of recalibrations within a sequence:
Calibration Table after Recalibration
Normal Report after Recalibration
If the sequence is done with bracketing:
Results of first cycle (ending previous bracket)

Signal 1: DAD1 B, Sig=240,4 Ref=off

RetTime [min]	Lvl Sig		Amount [ng/ul]	Area	Amt/Area	Ref	Grp	Name
7.446	1	1	8.24000e-3	237.95012	3.46291e-5			a-mangostin
		2	4.12000e-2	1223.90173	3.36628e-5			
		3	8.24000e-2	2472.22241	3.33303e-5			
		4	1.64800e-1	4899.56104	3.36357e-5			
		5	4.12000e-1	1.21819e4	3.38206e-5			
15.157	1	1	5.65600e-3	26.21124	2.15785e-4			garcinol
		2	2.82800e-2	145.44160	1.94442e-4			
		3	5.65600e-2	290.62256	1.94617e-4			
		4	1.13120e-1	591.90631	1.91111e-4			
		5	2.82800e-1	1490.40991	1.89746e-4			

Peak Sum Table

No Entries in table

Figure S22. Calibration curves: garcinol and xanthone (α-mangostin).

Method C:\CHEMSTATION\LCMS\1\METHODS\NARINGIN.M

Calibration Curves

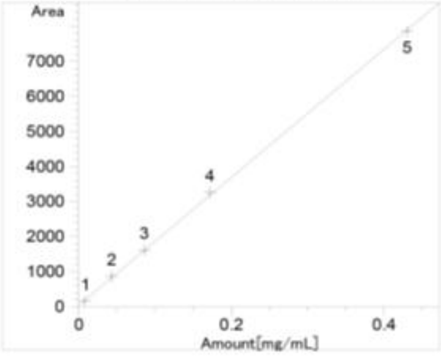

naringin at exp. RT: 8.848
DAD1 C, Sig=280,8 Ref=off
Correlation: 0.99987
Residual Std. Dev.: 54.27049
Formula: y = mx + b
m: 18257.58935
b: 39.76626
x: Amount
y: Area

Method C:\CHEMSTATION\LCMS\1\METHODS\NARINGIN.M

Calibration Table

Calib. Data Modified : 20/03/2019 3:54:27 PM

Rel. Reference Window : 5.000 %
Abs. Reference Window : 0.000 min
Rel. Non-ref. Window : 5.000 %
Abs. Non-ref. Window : 0.000 min
Uncalibrated Peaks : Separately calculated (see below)
Partial Calibration : Yes, identified peaks are recalibrated
Correct All Ret. Times: No, only for identified peaks

Curve Type : Linear
Origin : Included
Weight : Equal

Recalibration Settings:
Average Response : Average all calibrations
Average Retention Time: Floating Average New 75%

Calibration Report Options :
Printout of recalibrations within a sequence:
Calibration Table after Recalibration
Normal Report after Recalibration
If the sequence is done with bracketing:
Results of first cycle (ending previous bracket)

Signal 1: DAD1 A, Sig=210,8 Ref=off
Uncalibrated Peaks : using compound naringin
Signal 2: MSD1 TIC, MS File, Pos, Scan, Frag: 150, "Positive 150"
Uncalibrated Peaks : not reported
Signal 3: DAD1 C, Sig=280,8 Ref=off
Uncalibrated Peaks : using compound naringin

RetTime [min]	Sig	Lvl	Amount [mg/mL]	Area	Amt/Area	Ref	Grp	Name
8.848	3	1	8.60000e-3	152.43221	5.64185e-5			naringin
		2	4.30000e-2	862.50433	4.98548e-5			
		3	8.60000e-2	1615.30347	5.32408e-5			
		4	1.72000e-1	3255.11401	5.28399e-5			
		5	4.30000e-1	7856.55664	5.47314e-5			

Peak Sum Table

No Entries in table

Figure S23. Calibration curves: naringin.

https://doi.org/10.3390/foods15203720

```
Method C:\CHEMSTATION\LCMS\1\METHODS\QA-BP_PHENOLICS.M

=====================================================================
                        Calibration Curves
=====================================================================
```

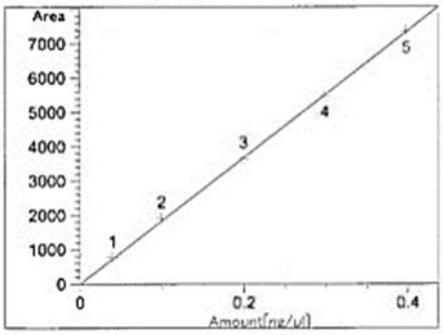


```
chlorogenic acid at exp. RT: 6.294
DAD1 C, Sig=330,4 Ref=off
Correlation:              0.99996
Residual Std. Dev.:      46.90613
Formula: y = mx
      m:    18455.99642
      x: Amount
      y: Area
```

```
Method C:\CHEMSTATION\LCMS\1\METHODS\QA-BP_PHENOLICS.M

=====================================================================
                        Calibration Table
=====================================================================

Calib. Data Modified   :      18/05/2017 4:04:15 PM

Rel. Reference Window :       5.000 %
Abs. Reference Window :       0.000 min
Rel. Non-ref. Window  :       5.000 %
Abs. Non-ref. Window  :       0.000 min
Uncalibrated Peaks    :       Separately calculated (see below)
Partial Calibration   :       Yes, identified peaks are recalibrated
Correct All Ret. Times:       No, only for identified peaks

Curve Type            :       Linear
Origin                :       Forced
Weight                :       Equal

Recalibration Settings:
Average Response      :       Average all calibrations
Average Retention Time:       Floating Average New 75%

Calibration Report Options :
    Printout of recalibrations within a sequence:
        Calibration Table after Recalibration
        Normal Report after Recalibration
    If the sequence is done with bracketing:
        Results of first cycle (ending previous bracket)

Signal 1: DAD1 A, Sig=210,8 Ref=off
Uncalibrated Peaks    :       using compound chlorogenic acid
Signal 2: MSD1 TIC, MS File, Pos, Scan, Frag: 150, "Positive 150"
Uncalibrated Peaks    :       not reported
Signal 3: DAD1 C, Sig=330,4 Ref=off
Uncalibrated Peaks    :       using compound chlorogenic acid

RetTime   Lvl   Amount      Area      Amt/Area   Ref Grp Name
 [min] Sig      [ng/ul]
-------|--|--|----------|----------|----------|---|--|---------------
  6.294  3  1 3.98000e-2  761.24487 5.22828e-5        chlorogenic acid
            2 9.95000e-2 1913.99133 5.19856e-5
            3 1.99000e-1 3644.70435 5.45998e-5
            4 2.98500e-1 5477.12842 5.44994e-5
            5 3.98000e-1 7361.42139 5.40656e-5
=====================================================================
                        Peak Sum Table
=====================================================================

***No Entries in table***
=====================================================================
```

Figure S24. Calibration curves: phenolic acid (chlorogenic acid).

Disclaimer/Publisher's Note: The statements, opinions and data contained in all publications are solely those of the individual author(s) and contributor(s) and not of MDPI and/or the editor(s). MDPI and/or the editor(s) disclaim responsibility for any injury to people or property resulting from any ideas, methods, instructions or products referred to in the content.

https://doi.org/10.3390/foods15203720
